# Supplementary material for: Chromosome compartment assembly is essential for subtelomeric gene silencing in trypanosomes
Source: Nat Commun. 2025 Nov 26;16:11669. doi: 10.1038/s41467-025-66824-3 (PMC12749359; doi:10.1038/s41467-025-66824-3)
Supplement: Supplementary file 1 — Supplementary Information [file 41467_2025_66824_MOESM1_ESM.pdf]

Supplementary Information

Chromosome compartment assembly is essential for subtelomeric gene silencing in trypanosomes

Luiza Berenguer Antunes[1#], Tony Isebe[1#], Oksana Kutova[1], Igor Cestari[1,2,*]

[1]Institute of Parasitology, McGill University, Sainte-Anne-de-Bellevue, QC H9X 3V9, Canada

[2]Division of Clinical and Translational Research, Department of Medicine, McGill University, Montreal, QC, H4A 3J1, Canada

* Correspondence: igor.cestari@mcgill.ca

[#] Equal contributions

Supplementary Figure 1. Analysis of the XLMS dataset. A) The graph shows the reproducibility of the *in vivo* PIP5Pase-V5 immunoprecipitation XL-MS dataset (*N* = 11). It shows the number of proteins reproduced per biological replicate. A reproducibility index (mean) of 6.1 (± 3.59), i.e., on average, a protein was detected in at least six different biological replicates. The data includes cross-linked proteins identified with a false discovery rate of 0.01. B) Reproducibility index of (as shown in A) for nuclear cross-linked proteins (*N* = 11). C) Frequency of cross-linked proteins detected by mass spectrometry (*N* = 54,157, cross-linked proteins). A protein was detected with an average frequency of 12.41 (±14.68). D) Distribution of cross-links per protein. It shows a mean of 13.23 (± 14.1) cross-links per protein. E) Top, diagram for the analysis of cross-links that likely occurred *in vivo* or after cell lysis. NE, nuclear envelope. Bottom, quantification of cross-links of bona fide nuclear proteins with other nuclear (Nuc-Nuc), cytoplasmic (Nuc-Cyt), mitochondrial (Nuc-Mit), endoplasmic reticulum/Golgi apparatus (Nuc-Golgi). Source data are provided as a Source Data file.

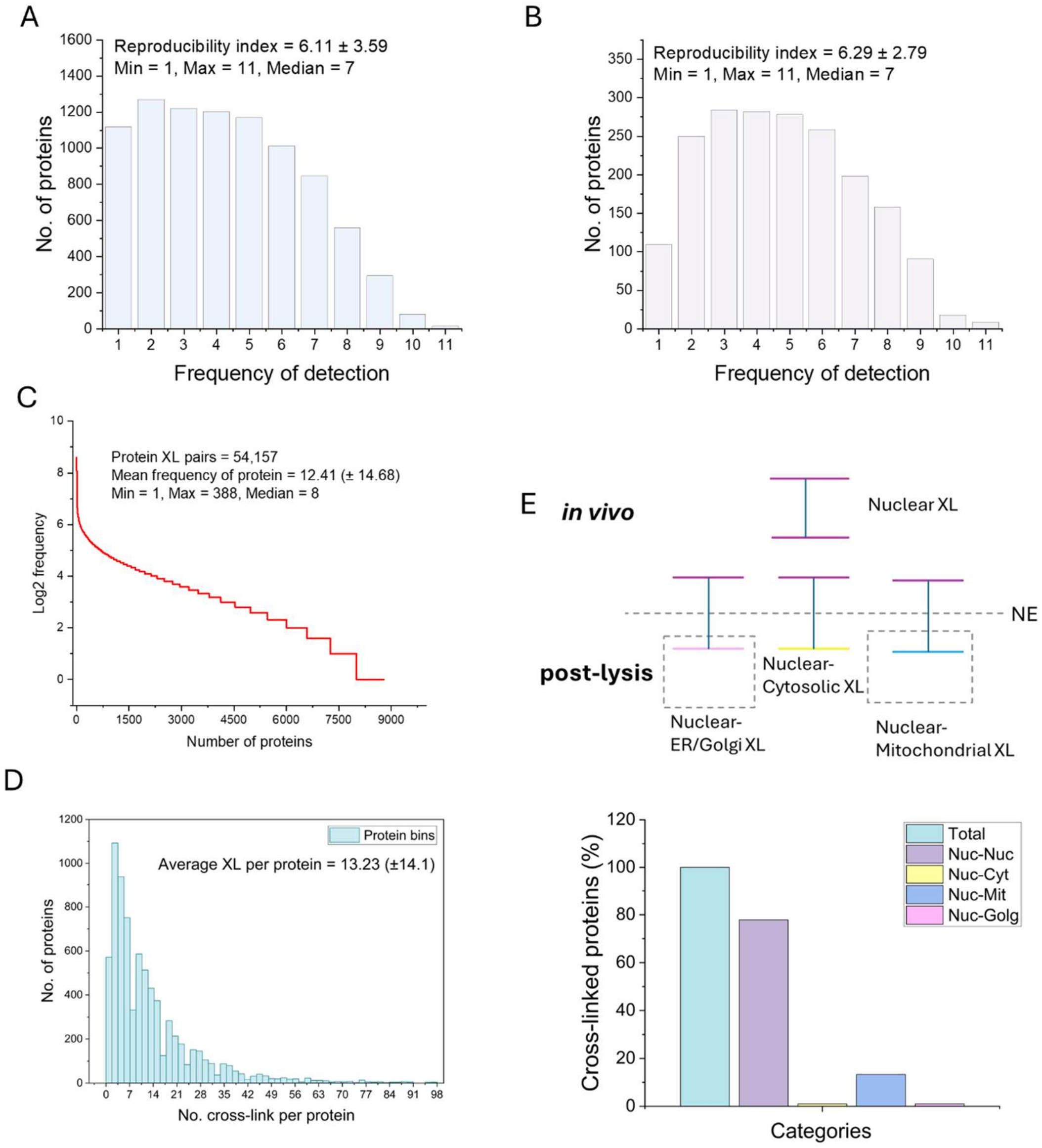

Supplementary Figure 2. PIP5Pase enrichment analysis. A) Enrichment analysis of PIP5Pase-V5 immunoprecipitations ($N = 11$) compared to immunoprecipitations from cells not expressing PIP5Pase-V5 ($N = 4$). Red dots indicate significantly enriched proteins (log2 fold-change ≥ 1, p-value ≤ 0.05). The description of a few enriched proteins is indicated by arrows. See Supplementary Data 2 for Gene ID and complete protein description. B) Data show enrichment of PIP5Pase immunoprecipitations comparing log2 fold-change with log2 mean of peptide spectrum match mass (PSM), which provides a relative indication of protein abundance. C) Network of PIP5Pase XLMS (N = *11*). Circles indicate proteins (colour-coded by functional groups), whereas connecting lines indicate cross-links. The large grey circles represent 30 top proteins identified in PIP5Pase-V5 immunoprecipitation and mass spectrometry without crosslinking (IPMS), as reported by Cestari et al. [13], also identified by XLMS in this work. See Supplementary Data 2 for source data.

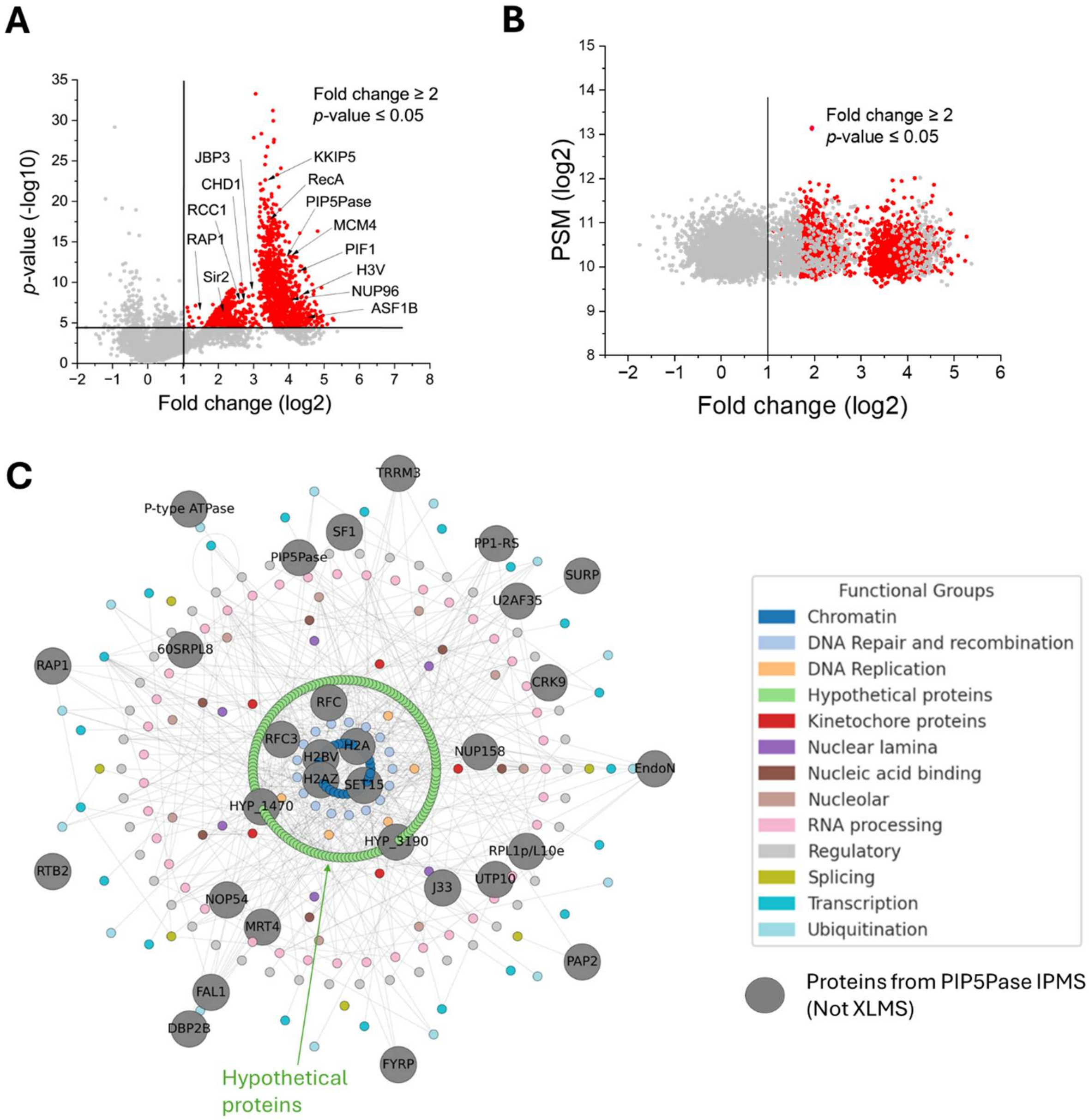

Supplementary Figure 3. Hi-C and compartment analysis. Hi-C matrix heatmap of each *T. brucei* Mb-long chromosome at 10 Kb resolution. Matrices were corrected using the Knight-Ruiz method. TADs identified from Hi-C data at 10 Kb resolution are shown. On the right, the diagram shows core gene (green) and subtelomeric gene (pink) regions. Left, RNA-seq of *T. brucei* SM427 strain. The data show bins per million mapped reads (BPM). RNA-seq was performed with Oxford nanopore sequencing. A mapQ = 30 was used to filter RNA-seq alignments. Below the matrix heatmap, it displays compartment analysis, where A compartments are indicated by positive values and B compartments by negative values. Note the correlation between subtelomeric regions with B compartments and core regions with A compartments. ChIP-seq of BDF2 show peaks at the transcription start regions and compartment boundaries; ChIP-seq of KKT2 shows centromeres; ChIP-seq of RAP1 shows its predominant distribution to subtelomeric, compartment boundaries, and centromeric regions. RNA-seq of *T. brucei* bloodstream forms expressing WT PIP5Pase compared to its catalytically inactive (D360A/N362A) mutant by nanopore sequencing shows derepression of subtelomeric regions and decreased expression of core regions. Hi-C data is the sum of three biological replicates. RNA-seq and ChIP-seq show the average of three biological replicates. For data on chromosome 9, see Fig. 3C.

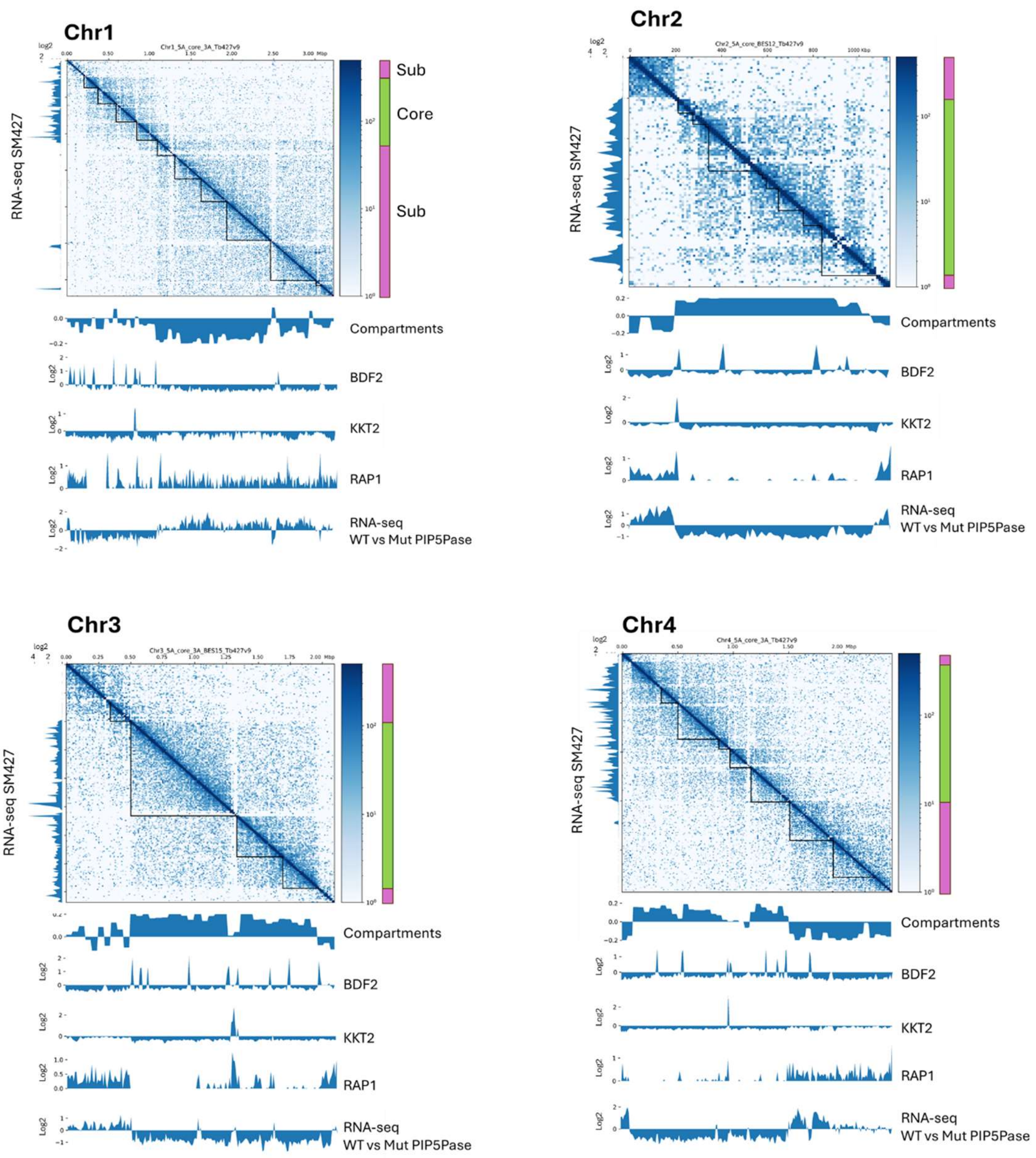

Supplementary Figure 3. Continued.

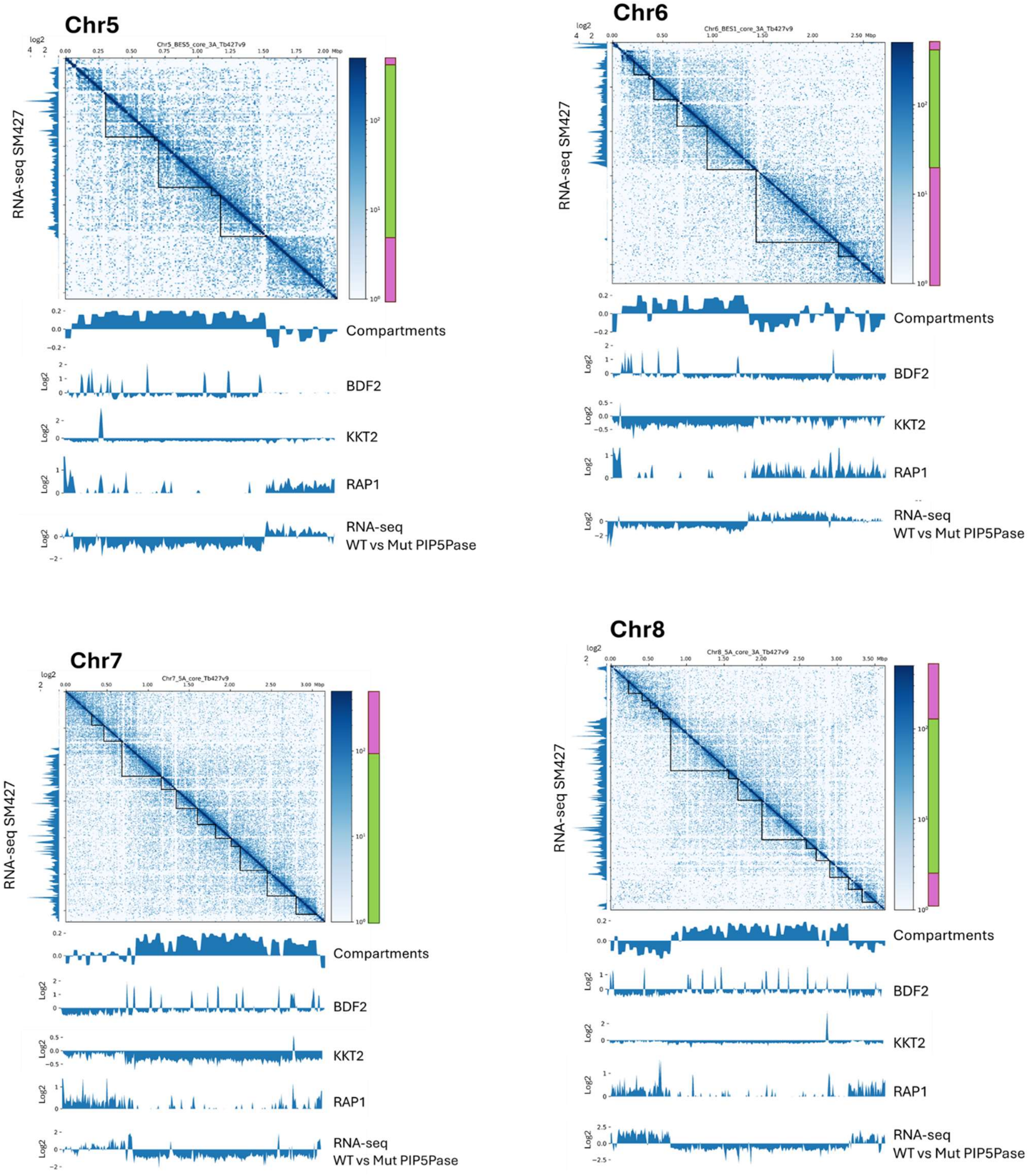

Supplementary Figure 3. Continued.

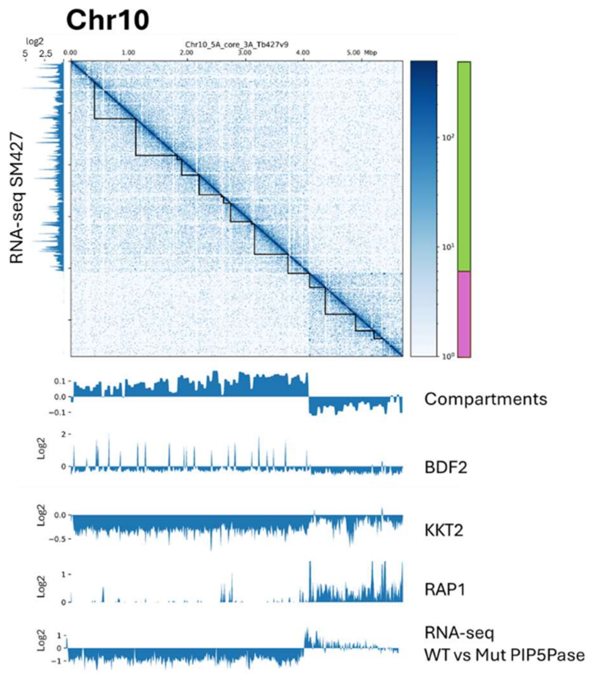

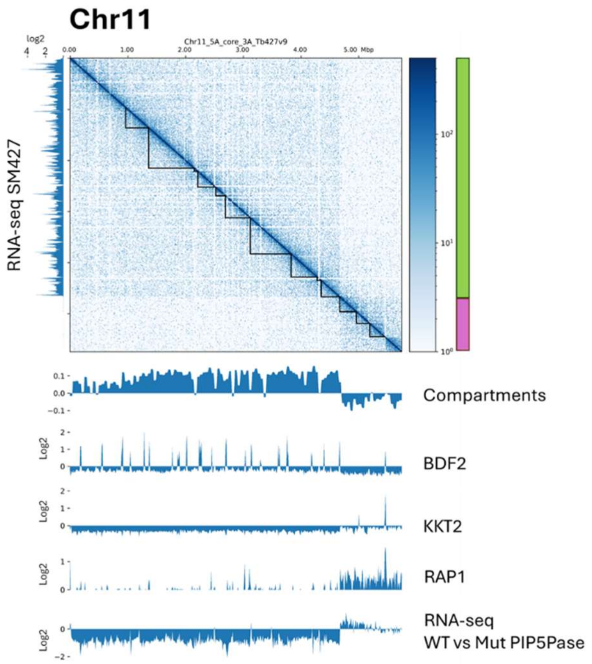

Supplementary Figure 4. Analysis of TADs by Pore-C and Hi-C. A) Heatmap of Pore-C (top) and Hi-C (bottom) interaction matrices at 100 kb resolution and corrected using the KR method, showing interactions between the 11 Mb-long chromosomes. B) Comparison of the amount of chromosome cis and trans contacts between Hi-C and Pore-C. C) Heatmap of Hi-C contact matrices for chromosome 9 at 10Kb resolution. Hi-C and Pore-C TADs are shown. Note sub-TADs, i.e., TADs within TADs, identified by Pore-C. D) Number of TADs identified by Hi-C and Pore-C searching a 1Kb resolution matrix. The same statistical parameters were used for both analyses. The *p*-value corresponds to the *p*-value threshold for the Bonferroni correction. The delta corresponds to the minimum threshold of the difference between the TAD-separation score of a putative boundary and the mean of the TAD-separation score of surrounding bins. The delta value reduces spurious shallow TAD boundaries, which can occur at the center of large TADs when the TAD-separation score is low. A comprehensive analysis of TADs at multiple matrix resolutions is shown in Supplementary Data 6. Source data for Figures 4B and 4D are provided as a Source Data file.

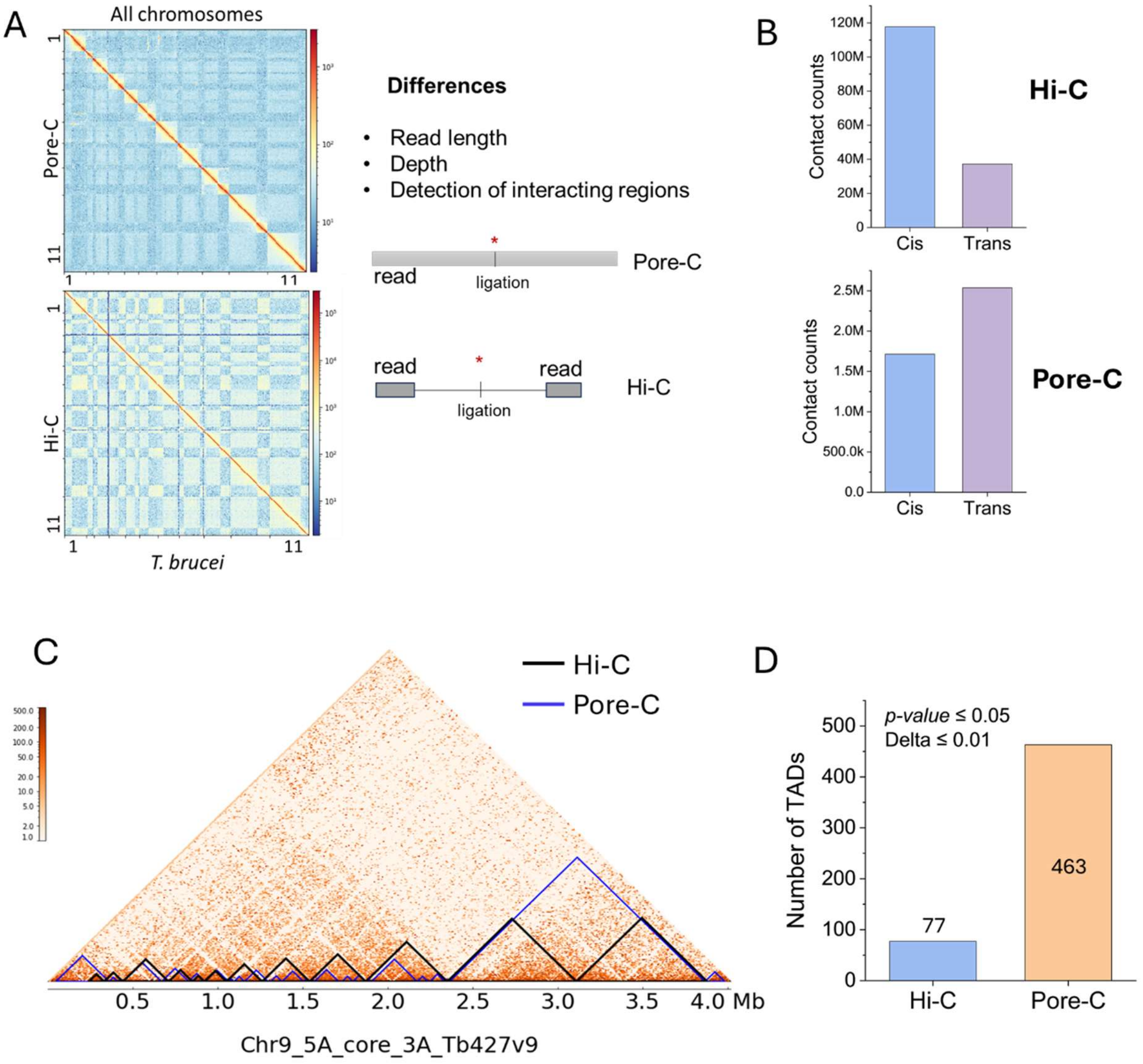

Supplementary Figure 5. Hi-C analysis of *T. brucei* SM427 Mb-long chromosomes. Hi-C matrix heatmap of each *T. brucei* Mb-long chromosome at 10 Kb resolution. Matrix was corrected using the Knight-Ruiz method. TADs identified from the Hi-C matrix at 10 Kb resolution are shown. Below the matrix heatmap, the graphs display compartments, where A compartments (core) are indicated by positive values and B (subtelomeric) compartments by negative values. ChIP-seq of RAP1 (primary reads mapped) are shown in red. ChIP-seq of BDF2, HAT1, HDAC1, ZCW1, SCC1, and H3V shows peaks at transcription start regions and compartment boundaries. ChIP-seq of KKT2 shows centromeres; ChIP-seq of RAP1 shows its predominant distribution to subtelomeric, compartment boundaries, and centromeric regions. Hi-C data is the sum of three biological replicates. ChIP-seq show the average of three biological replicates.

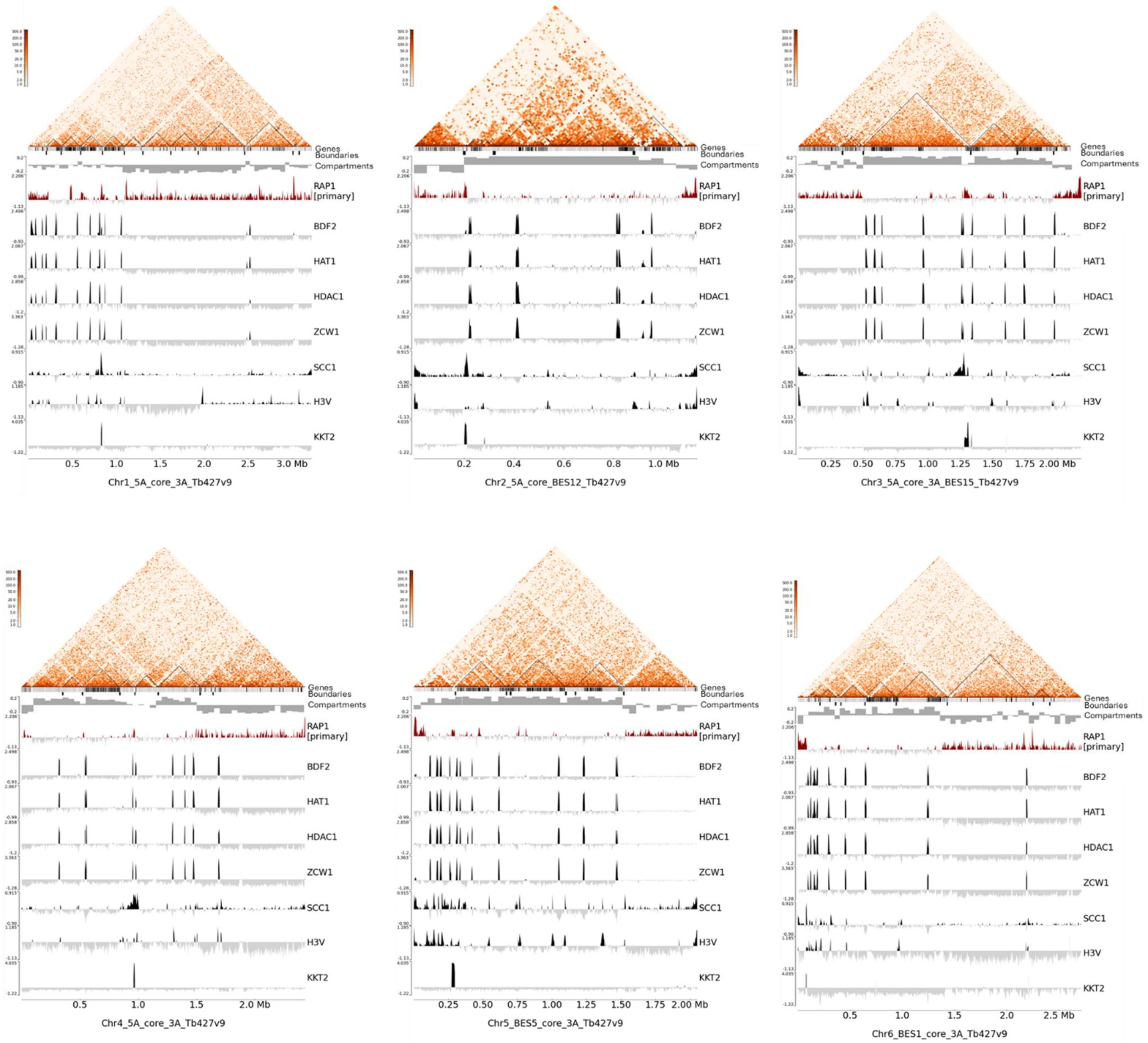

Supplementary Figure 5. Continued.

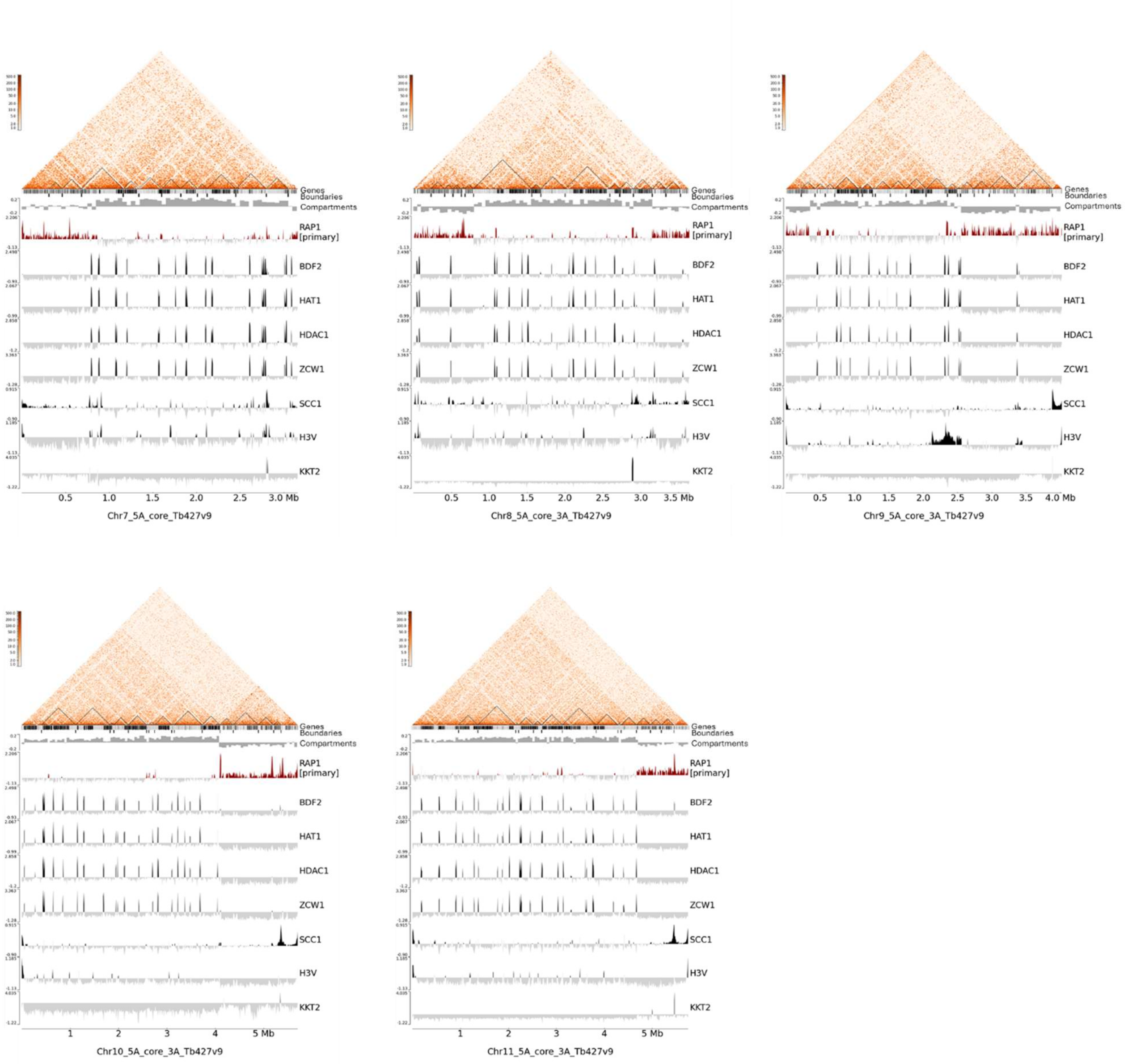

Supplementary Figure 6. Boundary motifs. Motifs identified in the boundaries of all 11 Mb-long chromosomes of *T. brucei* SM427. Motifs were searched in a ~10,000 bp boundary sequences bound by RAP1 using the Multiple Em for Motif Elicitation (MEME) tool using default parameters in the classic mode, except for site distribution set to any number of repetitions (anr) and number of motifs set to 100. The top shows a diagram of the boundary sequence with bars representing identified motifs. Below are sequences of identified motifs. The nomenclature used for nucleotides is indicated on the right (first page only). Each page shows results from a different boundary. Note similarities of sequences with the predominant (TTA)n(CAC) or TTTA and GGG/CCC repeats.

Name	*p*-value	Motif Locations
Chr1:1106718-1120840	0.00e+0	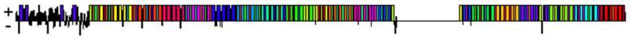

Motif	Symbol	Motif Consensus
1.		ATTATTGCTCTYATTAYAATATTCGTATATATACACTCACAAMACWWTCS
2.		ATTAYTSCTCTCATTATAATATTCGTATATATACACACTCACAACAC
3.		TAYTGCTCTCATTATAATATTCGTATATATACACWCWCAMMACWYTMYYS
4.		GTTACTGCTCTCATTATAATATTCGTATATATACACWCWCAMMACWCTM
5.		RTTAYTGCTCTYATTATAATATTCGTATATATACACWCWCAMMACWCTM
6.		ATTAYTGCTCTYATTATAATATTCGTATATATACACWCWCAMMACWHTM
7.		YTGCTCTYATTATAATATTYGYATAYATACACTCACAACAC
8.		GCTCTCATTATAATATTCGTATATATACACWCWCAMMAC
9.		GCTCTYATTATAATATTCGTATATATACACWCWCAMMAC
10.		GTATAYATACACTCACAACACTTTCCTCTTATTACTATCAT
11.		TTAYTGCTCTCATTATAATATTCGTATATATACACTCACAACACTATCG
12.		TAYTGCTCTYATTATAATATTCGTATATATACACWCWCAMMACWYTMYYS
13.		ATTATTGCTCTYATTATAATATTCGTATATATACACWCWCAMMACWCTM
14.		TATTGCTCTCATTATAATATTCGTATATATACACWCWCAMMACWHTMYYV
15.		RTTAYTGCTCTYATTATAATATTCGTATATATACACWCWCAMMACWHTM
16.		TACTGCTCTCATTATAATATTYGYATAYAYACACTCACAACACTWTCSTM
17.		TACTGCTCTYATTATAATATTCGTATATATACACTCACAACACTMTCGTA
18.		CGTATATAYACACACTCACAACACTMTCS
19.		TAYTSCTCTYATTATAATATTCGTATATATACACTMACAACACTTTCCTM
20.		TTATTACTATCATTAATATTATTATTAYTGCTCTTATTATA
21.		TCGTATTATTATTATTATCATTATGTTTA
22.		TCGTATTATTATTATTATCATTATGTTTA
23.		TMKTATTATTATCATTRTKWYTATYRTTAMTRYTMTTAT
24.		TTCCTCTTATTACTATCATTAATATTATT
25.		ATTATCATTATTATTATCATTAATATTAT
26.		CGTATTATTATTATTATCATTATGTTTAT
27.		TATTATTATTATTATCATTATGTTTATTG
28.		CTGYTRCTSCDWWTTGTTAGTKTHGGTCTKTGTTTWCTWWTTCDBTTKMC
29.		TTATYATTATTATTATCATTAWTATTAYT
30.		TTCCTCTTATTACTATCATTAATATYATT
31.		TCGTATTATTATTATTATCATTATGTT
32.		ATTATTATTAYTSCTCTYATTATAATATT
33.		TTATTACTATCATTAATATTA
34.		TTATTACTATCATTAATATTA
35.		TTATTACTATCATTAATATTA
36.		TCTTATTACTATCATTAATAT
37.		TTATTACTATCATTAATATTA
38.		AYCTSGBVGTGRCHG
39.		CATTATGTTTATTGT
40.		CSCAGC
41.		SGTGCC
42.		GGCGST
43.		SCGCTTSKKCTC
44.		SWCAASAGRGARCAC
45.		GAKCCTCYG
46.		GTGTKKSAGTSTG
47.		GWTGCC
48.		CCRTTYCTSTAWATCC
49.		GTKCCTAC
50.		CKGACTTC
51.		CTATCATCATC
52.		CSSTCC
53.		CATCTGWC
54.		GWCGAA
55.		GTGYWRAATSG
56.		GCWTWCAWAC
57.		TACYGA
58.		CYACAT
59.		AGTGGT
60.		CMCWRTACAWTC
61.		STCATMRC
62.		CATTATC
63.		CATTAWTATTATC
64.		CGTATWMTG
65.		TTTGST
66.		ATTCSW
67.		ATAAWG
68.		AAATAT
69.		ATAATATTA
70.		WAATAG
71.		CTATCA
72.		TACTKC
73.		TTGSTT
74.		CGWTAG
75.		ATKCST
76.		ATTATTATTAT
77.		ATSWTT
78.		ATAATAATAAT
79.		TTWTAA
80.		ATTAAT
81.		ATAATAAT
82.		ATAATAAT
83.		TTTTAT
84.		ATTTAT
85.		AATAAT
86.		TATAAT
87.		WTATAT
88.		TATTAA
89.		AWTCRT
90.		ATKATA
91.		TTCTAA
92.		ACWGAA
93.		ATTACT
94.		ATTWTT
95.		AAGYST
96.		TTWWCA
97.		TTTWSA
98.		TASAAR
99.		ATYAAS
100.		TGTTAC

Nomenclature

DNA

Symbol(s)	Name	Complement
A	Adenine	T
C	Cytosine	G
G	Guanine	C
T U	Thymine	A

Symbol(s)	Name	Matches
N . X	Any base	A C G T
V	Not T	A C G
H	Not G	A C T
D	Not C	A G T
B	Not A	C G T
M	Amino	A C
R	Purine	A G
W	Weak	A T
S	Strong	C G
Y	Pyrimidine	C T
K	Keto	G T

Supplementary Figure 6. Continued.

Name	*p*-value	Motif Locations
Chr1:3011606-3024991	0.00e+0	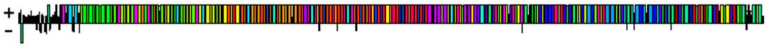

Motif	Symbol	Motif Consensus
1.		CGTATAWACACACTCACAACACACTCTCC
2.		CGTATAMACRCACTCACAACACACTCTCC
3.		CGTATAYACRCACTCACAACACACTCTCC
4.		CGTATAHACRCACTCACAACACACTCTCC
5.		CGTATAWACACACTCACAACACACTCTCC
6.		CGTATATACACACACTCACAAGACTCTCC
7.		CGTATAYACRCACTCACAACACACTCTCC
8.		CGTATAHACRCACTCACAACACACTCTCC
9.		CGTATAHACRCACTCWCAACACACTCTCC
10.		CGTATAWACACACTCACAACACACTCTCC
11.		CGTATATACACACTCWCAACACACTCTCC
12.		TATKAKTATTACTGCTCTTATTATAATATTCGTATAYACRCACWCAC
13.		ATTACTGMTYTWATTATAATATTCGTATAYACRCACTCACAAHACTMTYC
14.		CTGCTCTTATTATAATATTCGTATACACRCACTCACAATAC
15.		CTGMTCTWATTATAATATTCGTATACACGCACTCACAATAC
16.		TATTCGTATACACRCACTCACAATACTCTTCTATTATCATT
17.		TWYKYRTATACACRCACTCACAAKACTCTYCTATYATYATT
18.		TTCGTATATACACRCACTCACAATACTCTTCTATTATCATT
19.		TWMYKSYYTTATTATAAWATTCGTATATACACACWCWCAMMAYACTCTTM
20.		TTCGTATATACACACWCWCAMMAYACTCTYCTATTATYATY
21.		TACTGCTCTTATTATAAWATTCGTATAWACACACWCWCAMWACWCTYCY
22.		TACTGCTYTTATTATAAWATTCGTATATACACACWCWCAMMAYACTCTYC
23.		TATTACCATTATTATCATCATTATTATGAGTATTACTGCTC
24.		TATTACCATTATTATCATCATTATTATGAGTATTACTGCTC
25.		ATCATTATTACCATTATTATCATCATTATGAGTATTACTGCTC
26.		ATTATCATCATTATGAGTATTACTGCTCT
27.		TATTATYATTATTATTATYATTATCATTATTACTGATCTAATTATAAAAT
28.		ATTATTATCATTATTATTATTACTGCTTTTATTATAATATT
29.		TATYATYATTATYATYATTATTATSAKTATTACTGMTCTWATTATAAAAT
30.		ATTATCATTATTATTATTATTATTATYATTATTACTGMTCTWATTATAA
31.		ATTATTATCATTATTATTATTACTGMTYTWATTATAAWATT
32.		TATTATTATCATTATTACCATTATTATCATCATTATGAGTA
33.		ATTATTATYATTATYATTATTACTGATCTAATTATAAAATT
34.		ATTATYATYATTWTBAKTATTACTGMTCTAATTATAAAATT
35.		TATTATCATTATTATTATTACTGCTYTTA
36.		TATTATTATTATCATTATTACTGATCTAA
37.		TATYATYATTATKAKTATTACTGCTYTTA
38.		TATCATTATTATTATCATCATTATGAGTA
39.		TCTTCTATTATCATTATTATTATTATTAT
40.		ATTATCATTATTACTGATCTAATTATAA
41.		CTCACAACACACTCTCCT
42.		TATTATTATCATTATTATTAT
43.		TATTATCATTATTATTATTATTATTATT
44.		TATCATTATTATTATYATYAT
45.		GCCSACGG
46.		GSWDSCRMTWAVGMWSACTTY
47.		CTCTCC
48.		GSAACGRG
49.		ASCGGT
50.		TSCGTG
51.		GTRGGC
52.		SAGACAG
53.		CGTSTTC
54.		GKGAGTG
55.		TATTATTATCATTAT
56.		ACTGCT
57.		CTCTTC
58.		GGTTTS
59.		GTATATACAC
60.		TGKGCW
61.		GTTTASC
62.		TSCAGW
63.		ATCATC
64.		GWTTSAAG
65.		TTTGAG
66.		AAKTCG
67.		CTTTTMC
68.		CATTAWTTAC
69.		ACYATC
70.		TGATAA
71.		TGATAA
72.		ATTATC
73.		ATTATC
74.		ATTATC
75.		ATTATC
76.		TAAAAG
77.		TAAAAG
78.		ATATYG
79.		AAWAAC
80.		TATTATAAAAT
81.		TATTATAAAAT
82.		CAASWAC
83.		TGCMAT
84.		TAAAAG
85.		ATTATTATTAT
86.		TTATTATAAAA
87.		AATTATAAAAT
88.		TSATGA
89.		TATTATAAAAT
90.		ATTATTATATT
91.		ATTATC
92.		TATTATTAT
93.		CWTTTT
94.		TATTATAA
95.		TATTATAA
96.		TATTATAA
97.		TATTATAA
98.		TATCAT
99.		CATTAT
100.		TTATAATA

Supplementary Figure 6. Continued.

Name	*p*-value	Motif Locations
Chr2:1050262-1087616	6.94e-183	+ - 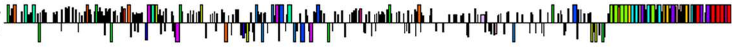

Motif	Symbol	Motif Consensus
1.		TTATTATTRTTATYATTATTATTATTRTTATYATTATTATTATTATTAT
2.		TATTATTGTTATCATTATTATTATTGTTATCATTATTAT
3.		TTATTATTNTTATCATTATTATTATTATTATTATTATTATTRTTATTATT
4.		TTATTATTRTTATCATTATTATTATTATWWWTTATTATTATTATTDTTAT
5.		TTATTATTATTATTATTATTATATTATTATTATTRTTAT
6.		GTGGGDRGWGDGTAGKRAWKSBTGCG
7.		TCADHVGGNSGMAHATCCAYCMVABTNHSGTAMAGKGCTSGVWGKWG
8.		TATTRTTATYATTATTATTAT
9.		AGTRRGWTYGTDCMATWGCASTGAGTAATSTGYT
10.		ATKGGAAGGGTWMAGYNTAMAMARGAAGK
11.		GAAGGGMTGCA
12.		CACWAGRRCWWBNTSTGYGKWWTGAWNTBWYWGAGCBCATGBWTRTG
13.		GATKTTDYGCCCSWMDWTGKWGRTGAWGS
14.		CGGYMCTKCTGAYAWMACC
15.		GGGWKKSAWKWWSGTGYVBCWWYYTCCGVAACAMCWYCCAS
16.		GCGCGC
17.		GTGGGAGCATG
18.		GGKRGGTGGWG
19.		AGGGWCTCWAA
20.		GAGGGTGC
21.		CACCGCAC
22.		CWACWAATSGWATWGCWTTGTACC
23.		GGAAGCACTG
24.		CCCAYCGG
25.		GCCCAC
26.		GCTCTGTTGAC
27.		GAAGCGG
28.		GGGGAG
29.		GGCGWGTG
30.		GGAGCAG
31.		CTTTTTCTTTTCTC
32.		GGAGCGG
33.		TTATATTATTATTAT
34.		TTTTTTCCCTC
35.		GTCACTGWGDTTTTA
36.		GACADMATSTCWAC
37.		GAGTTGGG
38.		GTGTGGG
39.		CCGCWTGC
40.		GCSTCGAC
41.		GCAGATGC
42.		GACCACTG
43.		GCGTGC
44.		CGTCGG
45.		GGGAGG
46.		GGGTAGC
47.		GTGCCAC
48.		GCAKTGTGCTG
49.		GCGACG
50.		GCCGAG
51.		GTGGCG
52.		GCWTGGGAC
53.		TTRTGATGCTGTAKA
54.		CAGSAAKCGATRCGC
55.		GTCGCG
56.		GTTATCATTAT
57.		AGAGTTAGAGCATTS
58.		GTGWTTTGCTCC
59.		TGGGGG
60.		CAASAAAGGAA
61.		GGATAAGGG
62.		GCTGCRTG
63.		GGGKATGC
64.		GGAGYTCC
65.		CTGTCGC
66.		TCCYTAAGGTTTTGA
67.		CCGATG
68.		TGTGCC
69.		CACAGG
70.		CATTATTATTATTDWTAT
71.		CGAGSAG
72.		GCKGTTTGSG
73.		CTGACACAG
74.		GCAKTTACCG
75.		CTTCCATG
76.		CACTGG
77.		CACAGWGATG
78.		GTGTGAWGAC
79.		CAAAGGWACC
80.		CCTTGAG
81.		GGSAGAAC
82.		GAACATSGG
83.		CCWCCKC
84.		GCTACG
85.		GCRMAGTCAC
86.		GSAAAGCAG
87.		GGAATGAC
88.		CTACACC
89.		GMGAMATGATGG
90.		CGAAAGG
91.		GGAAGTRTC
92.		GTGGAC
93.		CAGSCRWCTC
94.		CCMGAAWGTC
95.		TGCGTG
96.		GCRTGG
97.		CAGTGATAG
98.		CGCWCAC
99.		GTGTCG
100.		GGGSAAAC

Supplementary Figure 6. Continued.

Name	*p*-value	Motif Locations
Chr2:200721-214130	0.00e+0	+ - 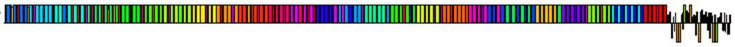

Motif	Symbol	Motif Consensus
1.		GYACAAAAGCRTGTTATTRCKTGTTTTATGYACAAAAGCGTGTTATTACG
2.		TGTGCAAAAGCATGCCATTACGTGTTTTATGCACAAAAGCG
3.		GYACAAAAGCRTGTYATTACKTGTTTTATGYRCAAAAGCRTGTYAATAYK
4.		KTRMAAAAGCGTGTTATTAHGTGTTTTATGTGCAAAAGCATGYYATTACK
5.		CAAAAGCRTGTYATTACKTGTTTTATGTRCAAAAGCGTGTTATTACGTGT
6.		MAAAAGCGTGTTATTAMGTGTTTTATGTGCAAAAGCRTGTYATTACGTGT
7.		CAAAAGCRTGTTATTACGTGTTTTATGTACAAAAKCGTGTTATTACGTGT
8.		CAAAAGCGTGTTATTAAGTGTTTTATGTRCRAAAKCGTGTTATTAMGTGT
9.		CAAAAKCGTGTTATTACGTGTTTTATGYACAAAAGCRTGTTATTAMGTGT
10.		CAAAAGCRTGTTATTRCKTGTTTTATGYACAAAAGCRTGYYATTACGTGT
11.		CAAAAGCRTGTTATTACGTGTTTTATGTRCAAAAGCRTGTYAWTATKTGT
12.		AAAAAGCGTGTTATTAAGTGTTTTATGTGCRAAAGCRTGTTATTAMGTGT
13.		MAAAAGCRTGTTATTRCKTGTTTTATGTRCAAAAGCRTGTTATTAAKTGT
14.		GTRCAAAAGCGTGTTATTAMGTGTTTTATGTRCAAAAGCRTGTTATTRCK
15.		GYRCAAAAKCGTGTTATTACGTGTTTTATGTGCAAAAGCATGTYAWTAYK
16.		GTACAAAAGCRTGTTATTAHKTGTTTTATGTDCAAAAGCGTGTTAYTACG
17.		GTRCAAAAGCATGTYATTRCKTGTTTTATGTACAAAAKCGTGTTATTACG
18.		AAAAGCRTGTTATTACGTGTTTTATGTRCAAAAGCRTGTTATTACKTGTT
19.		AAAAKCGTGTTATTAMGTGTTTTATGYACAAAAGCGTGTTATTAMGTGTT
20.		MAAAAGCGTGTTATTAMGTGTTTTATGTACAAAAKCRTGTTATTACGTG
21.		GTACAAAAGCATGTTATTGCTTGTTTTATGYACAAAAKCGTGTTAYTAMG
22.		MGTGTTTTATGTACAAAAKCRTGTTATTRCKTGTTTTATGTACAAAAGCG
23.		TGTACAAAAGCATGTTATTGCTTGTTTTATGYACAAAAKCG
24.		MKTGTTTTATGYACAAAAGCRTGYYATTACGTGTTTTATGTACAAAAKCR
25.		GTGCAAAAGCATGTCAWTAYKTGTTTTATGTRCAAAAGCRTGTYATTAHG
26.		RCTTGTTTTATGTACAAAAGCGTGTTATTAAGTGTTTTATKTWSAAAARS
27.		TGTTAHTACGTGTTTTATGTGCAAAAGCATGTCAATATTTG
28.		TTTATGTRCAAAAGCGTGTTATTAMGTGT
29.		ACAAAAGCATGTTATTGCTTGTTTTATGC
30.		TTTATGYRCRAAAGCGTGTTAYTACGTGT
31.		TTTATGTACAAAATCGTGTTATTACGTGT
32.		TTATGTRCAAAAGCATGYYATTRCKTGTT
33.		TGTTATTRCKTGTTTTATGTRCAAAAGCR
34.		TGTRCAAAAKCGTGTTATTAHKTGTTTTA
35.		TGTTACTACGTGTTT
36.		CVHYMYKCTGWDMTKGSWAGMGWCCCWSG
37.		RAAAAAGCRTGTTATTA
38.		CAGSATCGGS
39.		SCSTGTCCS
40.		GSWSMAAACAWCAGKSTTWCSGAGAC
41.		TGSCGG
42.		GCAGSAG
43.		CGGAASC
44.		CACSASSATRTGG
45.		GSGAWAGASC
46.		GAGAAGRAGC
47.		GACMASTGG
48.		GTGMCC
49.		GWGTGG
50.		GTSGARG
51.		GTTATTAAGTG
52.		CWCTWRGGYG
53.		CTCACWTS
54.		GSTATSMATTG
55.		GTGTTC
56.		GTRCAAAAG
57.		STCAAAG
58.		ATGTAC
59.		GTTTTATTTTG
60.		AGGAWA
61.		AATCAG
62.		GTTTTATTTYG
63.		TGTCAM
64.		GTTTTA
65.		GTTATTG
66.		GTGTTA
67.		TTATGT
68.		TTATGT
69.		TTATGT
70.		TTATGT
71.		TTATGT
72.		TTATGT
73.		TTATGT
74.		TTATGT
75.		TTATGT
76.		TTATGT
77.		TTATGT
78.		TTATGT
79.		TTATGT
80.		TTATGC
81.		TGTTTT
82.		TGTTTT
83.		TGTTTT
84.		TGTTTT
85.		TGTTTT
86.		TGTTTT
87.		TGTTTT
88.		TGTTTT
89.		TGTTTT
90.		TGTTTT
91.		TGTTTT
92.		TGTTAT
93.		ATTTTG
94.		TATGTA
95.		TATGTG
96.		TTKAGC
97.		TACTTC
98.		TAWCTS
99.		WTCSTG
100.		ATTTTG

Supplementary Figure 6. Continued.

Name	*p*-value	Motif Locations
Chr3:473866-531917	0.00e+0	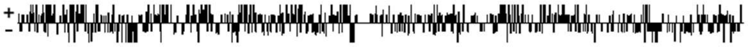

Motif	Symbol	Motif Consensus
1.		AATRATAATRATAGRADAGTGTTGTGAGTGTGTRTATACTAATATTATA
2.		TGHCTTCGKGAGMGTTBCCCYSCWCYGGCKHBGSCAKCANMMATCACCG
3.		TTTVTDRAATTTGCTAAAATWTGATATATTTTAACRYTTTT
4.		GGCGACTGKSGCWGABTSWCCCTTTTBTATTCATCCHAMC
5.		CAGCRGATGSVGSRAKARKCCAVCMACCC
6.		CSCWYTTTCTGTTKHTGTTSCTKKTKVWYYKBMTMCYSYYYCTGTYKCTG
7.		GACDRAACTDGCBAAAAGAAAGGAACAGGAGMHVAHTGCAAAG
8.		CDGHHASCGCAGCGGCHRCVGACGHMTKCVAARVNAACRSAMSGVAVAM
9.		TTCHAGTHTTCTYGTCAATAAGAAATTGG
10.		TAKWYTAWTTTGTTTATTTTATGKATATTADTTRNDDTTATDATRDTAAT
11.		AATWBRADYRYTTNARACAAYGRHRAGAATGCAATGRTARTAARTGAAT
12.		GASCCCWGAKATGCTATMGG
13.		CAVACTTYHBCAAWGATKTHARTTADMAATTTTTKTKTTTTGVTYTTTTT
14.		TGCAACCRGKGACGCAGCCSG
15.		CTCTCTATAATTTTTGTTGTAGCTMCGCA
16.		CCAWCMAWGCCAGGADCGCAGMANG
17.		TACMYYYTSSYCYCCACMCACAC
18.		AADCADCARAARRHAAGNARAAADWAYGBGDMAAGCACCAHGACARSAAA
19.		CTGATDGCTGCTGHBTTTATGDGTTTVGTAGHATTYTA
20.		GAVASAGAGTBCAGTGTRCAVVCCAAATRRDSTCTVTVCBTWATYCGCRG
21.		TGCCNGGGVSATCACTGTDGCMAKCGCGDDBANGSNTWNCACCGCAKTGS
22.		GVAANGCBASCGVCGACKGCDKCASCMCTVGYAGCMAHGVHASNCCAMM
23.		SGCTGKCGCYGCGGBGGCTC
24.		GTGCCDCMRCAGYGSHSMYKGBRSAASMCGS
25.		KYCTCATTKACCATDTCCTCCDCCTTTBNNHHRAKNTCTG
26.		CCCTGCAATHTTGCTTTCCATG
27.		YCAGTGTCHTCAVNYTTGTGCANCCACCKADNRCACTSADGYCTT
28.		ANGACRCACTSATCVTYMRHSNHAGTRCBBBCCAAACWCAAYTYCCTCAM
29.		CCCACAHCCTBCACAAMSRMAHHCCCCRG
30.		SCWCYCGCACCCATNCAAMCAAWMSNCCG
31.		CCCCCCCCCC
32.		GCAGCGGCCAG
33.		CSCAKCGCCSSCCRGGYTCCC
34.		AGGDACGMKCGSCASCRCKSG
35.		GCTACRCGGTTCCYCWGCKCG
36.		GSAGDGSWRCTGCSCCKGRCT
37.		SGAAGVGCTCGCTTRGGWSGG
38.		GGCTTGAGSGC
39.		GCAGCTGGTG

Supplementary Figure 6. Continued.

Name	*p*-value	Motif Locations
Chr3:1961826-1991538	0.00e+0	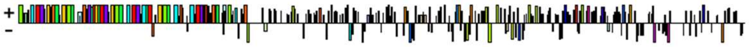

Motif	Symbol	Motif Consensus
1.		CARYTCCTCAAGBGTARYRAKGTTVGAGAGGGSYGYGACATCWVBHAACY
2.		KADRTYCARYACWYDAAGCYGTGGCAGBTYNCCAAGDVHNCCCABTCC
3.		RCAGYTGYYHAGATTCARYTCCTCCARTGCYGHAAGGBDWGAWATWDSDG
4.		CTTTGCAVHTCTCAAGAYTGAGYCTHACAARWGATTTRCHMKTCCCAA
5.		AGDNTKGSNACACGYAGYTGNKGAAGYKTWCCAAGHGCHTYCMADCC
6.		AYATCTGTYANYTTCTTRCAATGNGAAADGTTBARTNWYACMAGYG
7.		CASYASCGACGGCGVC
8.		AAGCACDGCAHRCDCMAMCAC
9.		AAGDNYCARTTCCTCCADHGTHNNAAYDTTRGABA
10.		YGAGGBGNTNASTGWTGGGGA
11.		GAGGGGGG
12.		CCTCCCGG
13.		TTGTGYAACCCCWWCGGAAGG
14.		GCGCAMCCARGGAGSC
15.		CTGAAGCACGC
16.		CATACGCGTGC
17.		TCTCBCBCATGTDNACDTCCTTC
18.		CAGGTSMSSGTGTGCAGYCCA
19.		GCTRCAGGCGG
20.		TCAGCDGCAACAACACYGNTVAHDRTGM
21.		GGGSATGAGGG
22.		CGGGTGCC
23.		CCCCATSCGC
24.		GTGAAGCGGKKTCCC
25.		GGCSGCAGWAACCA
26.		TCYACYGTGAAAMCWSMASDG
27.		GGMACCTMTCAGAMGCYCTTC
28.		TTGASSCCGTCTGAA
29.		CAGSCWGTGGATTTC
30.		GCSGKGAATGMCGGYTYCAAC
31.		CCTGCCGC
32.		GGAGAGGG
33.		CGGAGCKTTGSTWATGC
34.		CCCGTTTCCG
35.		CGAGCMACCCA
36.		ACSACACCAGC
37.		TGGCCAWGGTG
38.		CGSYAGTGGCC
39.		TTGCGYSCCGG
40.		GGWGCCRTMGGGTCA
41.		GCWGGCCRGACGT
42.		CGSCCCWTAYAACGC
43.		CGTGCSSTACG
44.		GSTGATGCCG
45.		GGCACC
46.		GTGTGGCAA
47.		GGAACATTRTKGCGC
48.		GTTGATTGRSCGTCT
49.		AGTGCCWTCACAC
50.		CCTGCTG
51.		TTCCARCCGTG
52.		CGCAGTG
53.		GTGGTGKACAC
54.		GCAGCRAAAGG
55.		CGAGAGYCACA
56.		GGATGAGRGGT
57.		TTCATCCKGCC
58.		CGCAGTSGTAA
59.		GTTGSATTCGG
60.		CCTTSCCTTTC
61.		CTCAGGTG
62.		AGTGATCSGCA
63.		GTCAGCAC
64.		ACACTGWGGTG
65.		TGYGVCCAVAGASTG
66.		CCTCGC
67.		CGCCTRTCC
68.		AGTCWCTSCACTYCC
69.		TGCTYTTTCTTTTT
70.		GSGAACAAAMGGWGC
71.		CCTCAWCWGGGRAAG
72.		GATRCAWGAAACSCWGCWGCA
73.		TGTGCAATACA
74.		GAAGGC
75.		GGGAAC
76.		GATGCTCT
77.		TCCAACTG
78.		TGARCAGTKTCTTCC
79.		GCSCACG
80.		TCGTTGTTG
81.		TGCTKCSACATCAAT
82.		AGATTCCACAGATAT
83.		CCGTGGC
84.		TGGCRTCASGC
85.		CACAGKSTCCG
86.		TCCCCT
87.		GTTGCTG
88.		TGCTTGACTT
89.		CAGTWCTCYGACATT
90.		TTTTCTGATGTTWGRRCARCWATCAAG
91.		TCGCTYYCTCC
92.		CCATWCGGCG
93.		GACCCAG
94.		GGCGGSAAAC
95.		GGCGCG
96.		TGCMCWTATKTCCCC
97.		TCAGTTGC
98.		TGAGGTTG
99.		CAGGARCMCC
100.		CAATAAAGTGTG

Supplementary Figure 6. Continued.

Name	*p*-value	Motif Locations
Chr3:1961826-1991538	0.00e+0	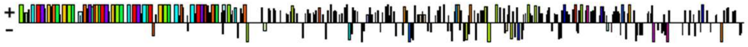

Motif	Symbol	Motif Consensus
1.		CARYTCCTCAAGBGTARYRAKGTTVGAGAGGGSYGYGACATCWVBHAACY
2.		KADRTYCARYACWYDAAGCYGTGGCAGBTYNCCAAGDVHNCCCABTCC
3.		RCAGYTGYYHAGATTCARYTCCTCCARTGCYGHAAGGBDWGAWATWDSDG
4.		CTTTGCAVHTCTCAAGAYTGAGYCTHACAARWGATTTRCHMKTCCCAA
5.		AGDNTKGSNACACGYAGYTGNKGAAGYKTWCCAAGHGCHTYCMADCC
6.		AYATCTGTYANYTTCTTRCAATGNGAAADGTTBARTNWYACMAGYG
7.		CASYASCGACGGCGVC
8.		AAGCACDGCAHRCDCMAMCAC
9.		AAGDNYCARTTCCTCCADHGTHNNAAYDTTRGABA
10.		YGAGGBGNTNASTGWTGGGGA
11.		GAGGGGGG
12.		CCTCCCGG
13.		TTGTGYAACCCCWWCGGAAGG
14.		GCGCAMCCARGGAGSC
15.		CTGAAGCACGC
16.		CATACGCGTGC
17.		TCTCBCBCATGTDNACDTCCTTC
18.		CAGGTSMSSGTGTGCAGYCCA
19.		GCTRCAGGCGG
20.		TCAGCDGCAACAACACYGNTVAHDRTGM
21.		GGGSATGAGGG
22.		CGGGTGCC
23.		CCCCATSCGC
24.		GTGAAGCGGKKTCCC
25.		GGCSGCAGWAACCA
26.		TCYACYGTGAAAMCWSMASDG
27.		GGMACCTMTCAGAMGCYCTTC
28.		TTGASSCCGTCTGAA
29.		CAGSCWGTGGATTTC
30.		GCSGKGAATGMCGGYTYCAAC
31.		CCTGCCGC
32.		GGAGAGGG
33.		CGGAGCKTTGSTWATGC
34.		CCCGTTTCCG
35.		CGAGCMACCCA
36.		ACSACACCAGC
37.		TGGCCAWGGTG
38.		CGSYAGTGGCC
39.		TTGCGYSCCGG
40.		GGWGCCRTMGGGTCA
41.		GCWGGCCRGACGT
42.		CGSCCCWTAYAACGC
43.		CGTGCSSTACG
44.		GSTGATGCCG
45.		GGCACC
46.		GTGTGGCAA
47.		GGAACATTRTKGCGC
48.		GTTGATTGRSCGTCT
49.		AGTGCCWTCACAC
50.		CCTGCTG
51.		TTCCARCCGTG
52.		CGCAGTG
53.		GTGGTGKACAC
54.		GCAGCRAAAGG
55.		CGAGAGYCACA
56.		GGATGAGRGGT
57.		TTCATCCKGCC
58.		CGCAGTSGTAA
59.		GTTGSATTCGG
60.		CCTTSCCTTTC
61.		CTCAGGTG
62.		AGTGATCSGCA
63.		GTCAGCAC
64.		ACACTGWGGTG
65.		TGYGVCCAVAGASTG
66.		CCTCGC
67.		CGCCTRTCC
68.		AGTCWCTSCACTYCC
69.		TGCTYTTTCTTTTT
70.		GSGAACAAAMGGWGC
71.		CCTCAWCWGGGRAAG
72.		GATRCAWGAAACSCWGCWGCA
73.		TGTGCAATACA
74.		GAAGGC
75.		GGGAAC
76.		GATGCTCT
77.		TCCAACTG
78.		TGARCAGTKTCTTCC
79.		GCSCACG
80.		TCGTTGTTG
81.		TGCTKCSACATCAAT
82.		AGATTCCACAGATAT
83.		CCGTGGC
84.		TGGCRTCASGC
85.		CACAGKSTCCG
86.		TCCCCT
87.		GTTGCTG
88.		TGCTTGACTT
89.		CAGTWCTCYGACATT
90.		TTTTCTGATGTTWGRRCARCWATCAAG
91.		TCGCTYYCTCC
92.		CCATWCGGCG
93.		GACCCAG
94.		GGCGGSAAAC
95.		GGCGCG
96.		TGCMCWTATKTCCCC
97.		TCAGTTGC
98.		TGAGGTTG
99.		CAGGARCMCC
100.		CAATAAAGTGTG

Supplementary Figure 6. Continued.

Name	*p*-value	Motif Locations
Chr4:949914-986834	0.00e+0	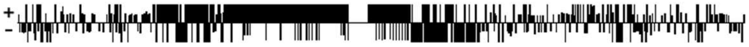

Motif	Symbol	Motif Consensus
1.		TACATGTTGCGTTGCTACACTGTTTTYGCACAATCATACGCATAAACRCG
2.		TACATGTTGCGTTGCTACACTGTTTTTGCACAATCATACGCATAAACRCG
3.		TACATGTTGCGTTGCTMCACTGTTTTTGCRCAATCATACGCATAAACRCG
4.		TACATGTTGCGTTGCTACACTGTTTTTGCACAATCATACGCATAAACGCG
5.		TACATGTTGCGTTGCTACACTGTTTTTGCRCAATCATACGCATRAACGCG
6.		TACATGTTGCGTTGCTACACTGTTTTTGCACAATCATACGCATRAACGCG
7.		TACATGTTGCGTTGCTACACTGTTTTTGCRCAATCATACGCATDAACGCG
8.		CGCAATAGTGCACAATTTGCACACAAACATGCMACATTGTGCATGTTATG
9.		CGCAATAGTGCACAATTTGCACACAAACATGCMACATTGTGCATGTTATG
10.		GCMACATTGTGCATGTTATGCAAGAATGCGTGTTTTACACC
11.		GCAATAGTGCACAATTTGYRMACAAACATGCAACATTGTGC
12.		GCAATAGTGCACAATTTGYRMACAAACATGCMACATTGTGC
13.		GCAATAGTGCACAATTTGYRMACAAACATGCAACATTGTGC
14.		GCGTTGCTACACTGTTTTTGCGCAATCATACGCATRAACGC
15.		GCAATAGTGCACAATTTGYRMACAAACATGCMACATTGYGC
16.		GCGTTGCTACACTGTTTTTGCACAATCATACGCATAAACGC
17.		GCAATAGTGCACAATTTGYRMACAAACATGCMACATTGYGC
18.		TACATGTTGCGTTGCTACACTGTTTTTGC
19.		CAATCATACGCATAAACGCGTATTTGACGTGAAAATACGCAATAGTGCAC
20.		TTGAMMMAAMMWKSCACATTGTGCATGTTATGCAAGAATGCGTGTTTTAC
21.		GCACACAAACATGCMACATTGTGCATGTTATGCAAGAATGCGTGTTTTAC
22.		TGCAAGAATGCGTGTTTTACACCAAAACACTTTACATGTTGCGTTGCTAC
23.		TGACGTGAAAATACGCAATAGTGCACAATTTGYRMACAAAC
24.		ATGTTATGCAAGAATGCGTGTTTTACACCAAAACACTTTAC
25.		GCAAGAATGCGTGTTTTACACCAAAACAC
26.		GCAAGAATGCGTGTTTTACACCAAAACAC
27.		GCAAGAATGCGTGTTTTACACCAAAACAC
28.		ATTTGACGTGAAAATACGCAATAGTGCAC
29.		CAAGAATGCGTGTTTTACACCAAAACACT
30.		CAAGAATGCGTGTTTTACACCAAAACACT
31.		TAYGCAAKARTGCRYRWTTTRCACMMAAACAYKYWACATK
32.		AAAAAAMAARAAHHAAAAAADACAMAATMAAVAAAMAMAAA
33.		TWCSSATAAACGCGTATTTGACGTGAAAA
34.		AKGGMYCYSGAAGAYCGCRYDCCRCAGBRCAVMYVCDGCK
35.		CVCGCBTTCGTTCWHYRCCAYGCCGSAGS
36.		CRAATRTGGCKGCGRAKSTKGTGSCGTMGKSRCG
37.		GCGGCGGDGTGGARCHSCRCGAWYBKAT
38.		AMARCACKCAACGCCTCAWCT
39.		GVTATTCGMKRDGYBDSCGSAGCGSRCCG
40.		TTCACMRVGCRGTGMTGVYCARCMTCTCS
41.		RTGYGCGKGCCRACAKCGKBVGCTSRCAC
42.		CAWTKCDWCASSGSCKTSCGSGSGWASATAAGKCMCKTKTGCDSMMAYTG
43.		SGGDADBGKCVCASMYKASMACCGSRATTTSKGCMGGGWC
44.		GTCHCAKWTVSCCGMCMGGRGGTTKRMWGVCMCCGMSGCVT
45.		CMSGACCTCKGKKTTAABAABSTYCWTTC
46.		CBKAAGAAKBDGRAVHGDTKGRAYGHSCACGARKYGGWGAAADSKMTGAG
47.		KTGGTTGGGGKGYRAGCGTYGCCARWGRA
48.		AWTGAADGGRRSAAMWGBTSGMMAWRCAVWGKTARMYWBCVGGVCGCWTG
49.		GSSWGTTTYKTGYYGKYCYGAARSYACWWRSCKMCRKKSCGBYGCA
50.		CCCMSSMKKCAACAWASSCAMMMCCAAAMGACAMMRCMATSCWSSARAA
51.		MTCAWRSGVDGDRCGVGRCGCAYCAHYGSATKTCATWKWWC
52.		TATTTTCACGTCAAA
53.		CRCAAAGMCAKCSAVAMCATCGWBGRY
54.		CWRTAWAAYARAWSVRWSAGCSRCTYTCSGTKTSWCATCCA
55.		YGYTKGTKGTSRYCKVRSRAAATMWACTCRKGGGYSVMCCG
56.		GYACTGGNGGTGGGSYNGYGAAAGNCACCGAATCCCTTCCT
57.		AKTGAAAAABDSGGYAHDYMGGMAMSDVHAWCATAYAGCAY
58.		CACCCGHAACTTCNKGCCGGDACNSAAGCCGCTGMTCCA
59.		AWGGAAWSSADTCGTACCTSCTGCACGGGAGASWYAYCM
60.		CTWRAMTYGAWAAGSRGRWTGCRAGRTGT
61.		ATTTTCACGTCAAA
62.		TATTTTCACGTCAAA
63.		TATTTTCACGTCAAA
64.		GTATTTGACGTGAAA
65.		TTGACGTGAAAATAC
66.		TATTTGACGTGAAAA
67.		ASRAWARAMMGAWAMAWCWRRDCAACAAA
68.		ACCACAAAAHGWCAYCAGSGBTTGRBGCBWYTCTTWGSTA
69.		GCTGTGRTWYHARGRACMASCYCYSCGGT
70.		CGGGBGAGSCCATTMRCCAACAGCKT
71.		CMGCAAAAAVGCCGTCMTGTGVTARMGGAHMWGCTYCCAAG
72.		CCVGCAGGDGVAMCHTDTCRTBGHTCCCTACRCGCCCCGGG
73.		CABCTTCGGTACTTYTCVMACNSCAGCCSGASSCMSRCVGHGATGTBACA
74.		AACTACADGACAGCSCMTCTWCMRCAHGAAAAYATTGTKTYMCCCAACA
75.		AAGCCKCWSRGCGCTKMMWAKWGAMWCCRYTTGGRYGAYGSCRYWTMMCC
76.		GCATTCATAGACCAAATCCACACGGAGAAATTTTCTCAGGACAGCCGACG
77.		CTAHTGYSRCACAYMAKCNCTSCWCVTGHCACRTWWWTGYAWNAACYGGA
78.		CCGKCGYWCRTGGRRGYGKCATTCATMSKYTWCRCRMMWGC
79.		GWDCAAYTGCATYMGACCVCASTTTGBAC
80.		CWSAYCCTCAGCCSAWGYGA
81.		CTGCYBCTSCGGTCGCCYDGAAWAACTTC
82.		CCTACATTCCGCCACGCCATACTTTGAAGAACCCGGC
83.		TAMTYATMCYCKSCCTTWRRGYWTTCGMMGTGASTTT
84.		CGGCGGCGCRCTKGCACGTG
85.		CAAKTRSHACACCTTTTTTGKMCYTCAGC
86.		ACAAAGVHATCGGTTCGCGMRCBAARCAG
87.		GAATRTKKDGTGTTGWKSCBYGCTGYKG
88.		GWTGNRCMKTTCTCTTCASCG
89.		ATTTGGCGCTATCGGCGCGATACACGAAG
90.		CSCKGARAGGYTTMGATCGGC
91.		CGGCGAAATTGCGAAGAATGTCGCTGGCA
92.		AGTGTTTTGGT
93.		ACTGTTTTTGC
94.		ACCAAAACACT
95.		TTCCACCGGTGGCAGGAACCG
96.		KMTCGCVCGSGCACTAHACVA
97.		GCGCAGTTACCATBAYTGACC
98.		AYASMCACHCACACACVCAC
99.		CACATTTCACGACCAAATGCAAAGAATGG
100.		CCATCAGAACCCATGCAACCG

Supplementary Figure 6. Continued.

Name	*p*-value	Motif Locations
Chr4:949914-986834	0.00e+0	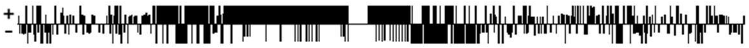

Motif	Symbol	Motif Consensus
1.		TACATGTTGCGTTGCTACACTGTTTTYGCACAATCATACGCATAAACRCG
2.		TACATGTTGCGTTGCTACACTGTTTTTGCACAATCATACGCATAAACRCG
3.		TACATGTTGCGTTGCTMCACTGTTTTTGCRCAATCATACGCATAAACRCG
4.		TACATGTTGCGTTGCTACACTGTTTTTGCACAATCATACGCATAAACGCG
5.		TACATGTTGCGTTGCTACACTGTTTTTGCRCAATCATACGCATRAACGCG
6.		TACATGTTGCGTTGCTACACTGTTTTTGCACAATCATACGCATRAACGCG
7.		TACATGTTGCGTTGCTACACTGTTTTTGCRCAATCATACGCATDAACGCG
8.		CGCAATAGTGCACAATTTGCACACAAACATGCMACATTGTGCATGTTATG
9.		CGCAATAGTGCACAATTTGCACACAAACATGCMACATTGTGCATGTTATG
10.		GCMACATTGTGCATGTTATGCAAGAATGCGTGTTTTACACC
11.		GCAATAGTGCACAATTTGYRMACAAACATGCAACATTGTGC
12.		GCAATAGTGCACAATTTGYRMACAAACATGCMACATTGTGC
13.		GCAATAGTGCACAATTTGYRMACAAACATGCAACATTGTGC
14.		GCGTTGCTACACTGTTTTTGCGCAATCATACGCATRAACGC
15.		GCAATAGTGCACAATTTGYRMACAAACATGCMACATTGYGC
16.		GCGTTGCTACACTGTTTTTGCACAATCATACGCATAAACGC
17.		GCAATAGTGCACAATTTGYRMACAAACATGCMACATTGYGC
18.		TACATGTTGCGTTGCTACACTGTTTTTGC
19.		CAATCATACGCATAAACGCGTATTTGACGTGAAAATACGCAATAGTGCAC
20.		TTGAMMMAAMMWKSCACATTGTGCATGTTATGCAAGAATGCGTGTTTTAC
21.		GCACACAAACATGCMACATTGTGCATGTTATGCAAGAATGCGTGTTTTAC
22.		TGCAAGAATGCGTGTTTTACACCAAAACACTTTACATGTTGCGTTGCTAC
23.		TGACGTGAAAATACGCAATAGTGCACAATTTGYRMACAAAC
24.		ATGTTATGCAAGAATGCGTGTTTTACACCAAAACACTTTAC
25.		GCAAGAATGCGTGTTTTACACCAAAACAC
26.		GCAAGAATGCGTGTTTTACACCAAAACAC
27.		GCAAGAATGCGTGTTTTACACCAAAACAC
28.		ATTTGACGTGAAAATACGCAATAGTGCAC
29.		CAAGAATGCGTGTTTTACACCAAAACACT
30.		CAAGAATGCGTGTTTTACACCAAAACACT
31.		TAYGCAAKARTGCRYRWTTTRCACMMAAACAYKYWACATK
32.		AAAAAAMAARAAHHAAAAAADACAMAATMAAVAAAMAMAAA
33.		TWCSSATAAACGCGTATTTGACGTGAAAA
34.		AKGGMYCYSGAAGAYCGCRYDCCRCAGBRCAVMYVCDGCK
35.		CVCGCBTTCGTTCWHYRCCAYGCCGSAGS
36.		CRAATRTGGCKGCGRAKSTKGTGSCGTMGKSRCG
37.		GCGGCGGDGTGGARCHSCRCGAWYBKAT
38.		AMARCACKCAACGCCTCAWCT
39.		GVTATTCGMKRDGYBDSCGSAGCGSRCCG
40.		TTCACMRVGCRGTGMTGVYCARCMTCTCS
41.		RTGYGCGKGCCRACAKCGKBVGCTSRCAC
42.		CAWTKCDWCASSGSCKTSCGSGSGWASATAAGKCMCKTKTGCDSMMAYTG
43.		SGGDADBGKCVCASMYKASMACCGSRATTTSKGCMGGGWC
44.		GTCHCAKWTVSCCGMCMGGRGGTTKRMWGVCMCCGMSGCVT
45.		CMSGACCTCKGKKTTAABAABSTYCWTTC
46.		CBKAAGAAKBDGRAVHGDTKGRAYGHSCACGARKYGGWGAAADSKMTGAG
47.		KTGGTTGGGGKGYRAGCGTYGCCARWGRA
48.		AWTGAADGGRRSAAMWGBTSGMMAWRCAVWGKTARMYWBCVGGVCGCWTG
49.		GSSWGTTTYKTGYYGKYCYGAARSYACWWRSCKMCRKKSCGBYGCA
50.		CCCMSSMKKCAACAWASSCAMMMCCAAAMGACAMMRCMATSCWSSARAA
51.		MTCAWRSGVDGDRCGVGRCGCAYCAHYGSATKTCATWKWWC
52.		TATTTTCACGTCAAA
53.		CRCAAAGMCAKCSAVAMCATCGWBGRY
54.		CWRTAWAAYARAWSVRWSAGCSRCTYTCSGTKTSWCATCCA
55.		YGYTKGTKGTSRYCKVRSRAAATMWACTCRKGGGYSVMCCG
56.		GYACTGGNGGTGGGSYNGYGAAAGNCACCGAATCCCTTCCT
57.		AKTGAAAAABDSGGYAHDYMGGMAMSDVHAWCATAYAGCAY
58.		CACCCGHAACTTCNKGCCGGDACNSAAGCCGCTGMTCCA
59.		AWGGAAWSSADTCGTACCTSCTGCACGGGAGASWYAYCM
60.		CTWRAMTYGAWAAGSRGRWTGCRAGRTGT
61.		ATTTTCACGTCAAA
62.		TATTTTCACGTCAAA
63.		TATTTTCACGTCAAA
64.		GTATTTGACGTGAAA
65.		TTGACGTGAAAATAC
66.		TATTTGACGTGAAAA
67.		ASRAWARAMMGAWAMAWCWRRDCAACAAA
68.		ACCACAAAAHGWCAYCAGSGBTTGRBGCBWYTCTTWGSTA
69.		GCTGTGRTWYHARGRACMASCYCYSCGGT
70.		CGGGBGAGSCCATTMRCCAACAGCKT
71.		CMGCAAAAAVGCCGTCMTGTGVTARMGGAHMWGCTYCCAAG
72.		CCVGCAGGDGVAMCHTDTCRTBGHTCCCTACRCGCCCCGGG
73.		CABCTTCGGTACTTYTCVMACNSCAGCCSGASSCMSRCVGHGATGTBACA
74.		AACTACADGACAGCSCMTCTWCMRCAHGAAAAYATTGTKTYMCCCAACA
75.		AAGCCKCWSRGCGCTKMMWAKWGAMWCCRYTTGGRYGAYGSCRYWTMMCC
76.		GCATTCATAGACCAAATCCACACGGAGAAATTTTCTCAGGACAGCCGACG
77.		CTAHTGYSRCACAYMAKCNCTSCWCVTGHCACRTWWWTGYAWNAACYGGA
78.		CCGKCGYWCRTGGRRGYGKCATTCATMSKYTWCRCRMMWGC
79.		GWDCAAYTGCATYMGACCVCASTTTGBAC
80.		CWSAYCCTCAGCCSAWGYGA
81.		CTGCYBCTSCGGTCGCCYDGAAWAACTTC
82.		CCTACATTCCGCCACGCCATACTTTGAAGAACCCGGC
83.		TAMTYATMCYCKSCCTTWRRGYWTTCGMMGTGASTTT
84.		CGGCGGCGCRCTKGCACGTG
85.		CAAKTRSHACACCTTTTTTGKMCYTCAGC
86.		ACAAAGVHATCGGTTCGCGMRCBAARCAG
87.		GAATRTKKDGTGTTGWKSCBYGCTGYKG
88.		GWTGNRCMKTTCTCTTCASCG
89.		ATTTGGCGCTATCGGCGCGATACACGAAG
90.		CSCKGARAGGYTTMGATCGGC
91.		CGGCGAAATTGCGAAGAATGTCGCTGGCA
92.		AGTGTTTTGGT
93.		ACTGTTTTTGC
94.		ACCAAAACACT
95.		TTCCACCGGTGGCAGGAACCG
96.		KMTCGCVCGSGCACTAHACVA
97.		GCGCAGTTACCATBAYTGACC
98.		AYASMCACHCACACACVCAC
99.		CACATTTCACGACCAAATGCAAAGAATGG
100.		CCATCAGAACCCATGCAACCG

Supplementary Figure 6. Continued.

Name	*p*-value	Motif Locations
Chr5:1459432-1476976	0.00e+0	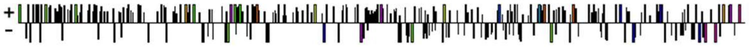

Motif	Symbol	Motif Consensus
1.		CACACACACACACA
2.		AGGGGCSCYTGCCCC
3.		CWGCAGAANARRSAAATTBCGATMTGCSAGDNTCMRVAAACGGCWVCRNG
4.		GACCCCCGGCC
5.		YCTKGATTTHCTHCCCHTCDVCTTCHTKYNCCHGGAG
6.		AGCGACCRCCYGMG
7.		CCSCCGSCRCTGCSTCCAG
8.		CCWACDVHTCCDCHSGCRDCRGACCCACCACAHBAARCAAT
9.		TGAGABTHGBTATVTGTGCTGTGKCTHWCTKANSTKT
10.		CGTCBTGDTTTCRARCCYGTKTCWCRAVA
11.		CAMCACWTGGNTATGGAAGS
12.		CCGTGCCGTG
13.		GCBGAGACAATGCGG
14.		TKKGTGGTTHDGTTGATGWTNTTTNKGNTGTATTTKTKGY
15.		TGGBDCGANTTSCTRGTGCVVBGTSGAVDBGGTGKDWTGHWG
16.		CCCMCCCCCTG
17.		CAGSGGGCCC
18.		GASGGGCGCC
19.		KTSAVTGGTTGBASDCGBGTDTTTGCCGT
20.		GSAGCKTVTGGBWTCTCHBCTCYADNTTK
21.		TATCTCCTTNTCYYTYWSDTSMNHAAVCDMYTCCAHTTT
22.		GTTCTTSCTTCBCCTCTTVGDMRCSVTG
23.		CTGCGCSCACC
24.		CCACCAAGGG
25.		GGCGGTGAC
26.		CGGAGCTCC
27.		GCCGATACTGC
28.		CGCACGTGYGRSCGC
29.		CAAVAAAAWAAAADRADTAA
30.		GCWCAACCWACNSACA
31.		GRKWACAWRGAGGKRAVTGSG
32.		TCGTCGBNCGTATGC
33.		ACDCCRGTVGAGGCR
34.		GCTRCCCCTGC
35.		CCATCCACTGKGRCAAKCTC
36.		CCGAAKTCSAGCGAG
37.		CCACCACC
38.		CTCGCRTGGCWAWGGTG
39.		CGGMAGMGTGCTG
40.		AGGCGGSASCGAA
41.		GCTSCTCSCARACGG
42.		CTCTMTCACCTGATC
43.		CTCACGAGTG
44.		CCRCGCACC
45.		TACGCACAYCCTC
46.		CGTTCTSCRATGCCT
47.		AGCCSAGTGSTTGTA
48.		AGGATCCTCMACC
49.		CGRCGCAGAGAA
50.		GTBGCWTCTCCTBTTTACCCMABGVCYNT
51.		GTGGGC
52.		TGTTYGSSGAAAKCMKCTTCACSRCSCC
53.		CGAAAACAGC
54.		SARAAHAVAMWGANGAATVCADANMGDAAAYKGVAVAAT
55.		ACCTTTTCC
56.		GACAACGAAAACGVCACC
57.		CATGTTKCGHCTCCA
58.		AGTGAGGCATT
59.		CGTDAAGGAAVAGCA
60.		CGGGGA
61.		CCACCAC
62.		CCTTCAGTGT
63.		CCCAATGG
64.		GTGTGTGRGG
65.		CTGMGCAGMAGCKCG
66.		GCCGARGCGG
67.		GGCGTCTTGG
68.		GGGCTGCWCC
69.		GGCMSGCCTCG
70.		CGTTTCTTCRC
71.		TCTGGAAGCKSGYCG
72.		GGGTGRARGGTAGSC
73.		CATCCSGCYGATG
74.		CACGAGAAACA
75.		CTNTTTGGGATTGTTNASDTTGTTCTTT
76.		AAAASAWCARCAAAAWNANCATTSAGAAT
77.		CGAAACGCSSTCT
78.		CCCCCA
79.		CCACTGCCG
80.		CGGCYGTTCAG
81.		TCCCCA
82.		GTCCCA
83.		GTGGATG
84.		GAGGAMGCAA
85.		CCTASACACCA
86.		TGCAMCCTC
87.		GCAGCMTGT
88.		CCGRGGCATCT
89.		CCTCYGCC
90.		CCCAATGGGC
91.		CCYACTGGACC
92.		CAGCTCCATC
93.		TCTCACSCTGC
94.		CSAACGCGAAG
95.		CACCGC
96.		CCGTCGC
97.		GGTCAGTCWCC
98.		CCAAAMAAGATAGCG
99.		AAGKGSGTGTGAGTKACA
100.		GACGGWGC

Supplementary Figure 6. Continued.

Name	*p*-value	Motif Locations
Chr6:1236382-1255448	0.00e+0	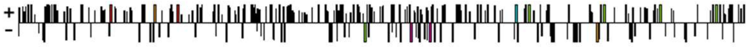

Motif	Symbol	Motif Consensus
1.		AGYGTBTGTGTDTGTGTGTDTGTGGARG
2.		ARGGGTGGCAGTGGCYBGBTCCCCCCCCG
3.		TCCAMAAGAGCCTC
4.		GCGGGAGGCNGCCGA
5.		AAGAGRAGACAARAAGAAGAA
6.		CCTCCAGGTCHGGSTTCTYCT
7.		SCGACCACKCTGAHGNABWAAADG
8.		TGTGCHGCGVSGCCGSCVVVAVCCAWVAGCHGVGSBCGACA
9.		CGCSDASCSKGKCAACMGC
10.		GCGACSGGCCYCSACGC
11.		AGCAGCWAAMARGGSMGC
12.		AAHCCCTTNGTGTCWVGWGG
13.		CGGCGTCWCCCC
14.		CCGCGCGG
15.		GTGTCGCAGCC
16.		TGTTGTGGABGAGTG
17.		CAATACCCTHCATBTNCCWSCBHTGBATBVGCDGVBGA
18.		CCCCTGCCSC
19.		CCGGGTGTCGSGG
20.		GTGVSATACVCGCABYCBTTTCWWTBTT
21.		ACSGGSACGCCC
22.		AGGWGKGCSGTSWGCGSCGGA
23.		CSCCTGCAGYGGA
24.		CGCGAGC
25.		GCCGCSGCSTG
26.		CGCTCCAGTTG
27.		CWCCYTTGYCACVMT
28.		GTTCTTTGTTCT
29.		CCYBCCGTGTTGTKHHNTCTNVATCGGC
30.		CCACCMKGTGSCGCAC
31.		GCGGYTGCMTSTGGRAGKGTG
32.		AASCCARCKCATCCASWTYGCCCC
33.		GGCGAWKGSKCTGCAKTTGCC
34.		GSAASGGGCAGACG
35.		CTTSGACATCACAGC
36.		GANVWARAAADGDGTVWBAAHTTBCTNAASRAMAAWAC
37.		GGGGGAAAAMA
38.		AAKAAATAAAASCAA
39.		YGCTTACCTYTWWCCADYSTTTTYYTTCT
40.		MGCDGHWGAAAAHGBTKMASGCNHAAAASNTGVWGATDANGTTDGNC
41.		GAACGCTGCTG
42.		GTGTTGGKCTGCC
43.		GCCRTTCCTCCTSGT
44.		TAGCACACTGTGSRGAAG
45.		CGAAGGTYGGCMSASGC
46.		GCTSGCACGTG
47.		CAAAGGGMTSTCCYTTTGG
48.		TGCAAGHKGCTTCCG
49.		CAKCAASSATGTTGG
50.		GGCAGCYGTCAMCAG
51.		CTCSTCGYGGTGGAA
52.		CCTGGAAGCG
53.		GSGCACWCAGGCAAT
54.		GGGGARSGKGTGTGG
55.		GCMGCTSGAASGGCA
56.		TCGYTCATGAGCTTC
57.		TCGACTCCTCAT
58.		AGGGAACGAAARGAG
59.		GTTGAGGGAATAG
60.		CGGTGRTGGAG
61.		GSSACSSGCMMTGGACTGAG
62.		CCCKCGTTTCTCT
63.		GGACMCTSGCAYMWCGTTGAG
64.		AACCCACSAACRSKGCTGG
65.		TCSCTGAACSSACTWCTGG
66.		TGATGCWGGGSC
67.		AWCHCBCTBTTTCTNCTGCHTTTSYTCC
68.		CCGCACC
69.		CGTKCGACCACWGSG
70.		CCCCAGTTAC
71.		GGCAAGGCG
72.		CACGCAGGG
73.		GGCGCTTTTGC
74.		GCCAMTGCASTGTCA
75.		GGAATAGKGATCGAA
76.		CCTGAARGWAGCGG
77.		CAACCCC
78.		GGAGACGG
79.		CATAAACGSGTSACG
80.		GGAAAGCG
81.		CCAASGGSAGMAGG
82.		CSACGWGCTACC
83.		TGYTGTTGCCWCKCATGGT
84.		ACTSTGAGGCWKCGWSTMGCG
85.		GTCACAGYGC
86.		GGAASTAATAACWGCWGTGC
87.		CACACACTG
88.		CAAMSCTCGKCTTACMAASSSTCGWACT
89.		CACCGTG
90.		TTSGTCTACCRTCKTTGTTTT
91.		CTGCCCC
92.		TTCGCYCATCC
93.		TCGGKGACACA
94.		CCSCAACYCMMGAACATC
95.		CTGCCCG
96.		GGTGVTWTBCTTATBGGAAHG
97.		GACAGCMCMKTAASTSGCG
98.		TGAAGAAGACT
99.		GCGACYSWCTGCTA
100.		CAGGACATT

Supplementary Figure 6. Continued.

Name	*p*-value	Motif Locations
Chr7:790832-892950	3.07e-249	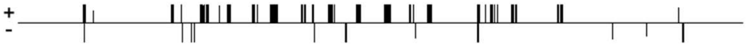

Motif	Symbol	Motif Consensus
1.		ATAAKGATAGRAGWGTGTTGTGAGTGTGTATATACRAATAT
2.		TGCAAATGGGADGGMRAARMWTGCAAAGATTCCAGTWTTCTTGTAAATAA
3.		TDATRRTRATAATAATRATAATRATAGGAGDGTGTTGTGAGTGTGTGTAT
4.		RAGGAARATRGRATATGCRCANBTTCAAACACAAACATKADDATGGDVAT
5.		GATGAYACKGSGKAYDGSACSCTGGCACS
6.		TTTTTCTCAATTTTATGAAATTTATAAA
7.		TYAYACACAAGGAGATGATGGAGGKGWBA
8.		AAATTTGATATRTTTTAAYACTTTBTRAHDAAAATVTKMAAAWTATTRWA
9.		RAAACYGCTTRMARYANYGCATATRTGARCCRBYAGAGRAGYTTTWTRWA
10.		ATGACAGTGRACYVTAGGTGTAAAGTANTATKGTDATADGGTATRAAAAA
11.		MTCTYTDDCYAAMAKCWTCTYCYDGHRKVAHACBCHATHTTAGCRRYGGT
12.		SAGSVAKCAGCABYBTYTTSMTGGGRVACMST
13.		AKRGRRAGANAVAGTCCAGTGTRCAAGGVAAATRGAG
14.		TTTAATHTRRTGGYGAATACTAATRTCCAGYTKTRAAGAAG
15.		AATRGAAAGGGTGCRATGGGA
16.		ACKTTGAGGTGGCCGKCRYCVMCRGGGT
17.		CYGAYGGCGCKGCGC
18.		GGGGGGGGGG
19.		GAATGCAARCCYAAASCAGGARCAGMAAACACARCAGC
20.		GAAATTNGCTCTGABDGCTDCTGCTTTTATRAGTTTGRTDDYATTTTAR
21.		TWTDTTTTTWATVDATAYTAVHTANNKTTATAATAGTAATRGTAATAATA

Name	*p*-value	Motif Locations
Chr7:3020739-3094115	0.00e+0	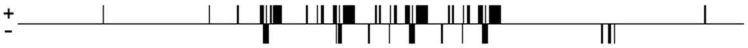

Motif	Symbol	Motif Consensus
1.		AACCGCTCCGTCAGCAAACCGGCGGTCGGCTCCCGCGGAGGCACACGGTT
2.		GAAGGTCGGTATCGTAAAGGATGCCTCCTCCGCACCAACAGGAGCTCGGG
3.		CATGCAGGCTCAGGCCCGTGTCCTCCGACTCTCCTCAGTAGCATTCGTGG
4.		GGCAGCACGCACGGAGAGTGTTGGTAACGGTGGTCAGGTGCTGATGACGC
5.		GGAAGTGGCGGTGTCCCGAGGCTTTTGGGGACCACATGAACTGCCCTTTC
6.		GGATAAGAGSCATGGGAGCCGCCAGGGCGTTGTCCCTTCAAGACGCACGG
7.		GCTACACTGTGCCTGTTCCATTGGAAATGCTTCCGTATCGGT
8.		TGKRRMCKKAWCKGASTGAKASCAWAASGGASMAGWGMAAGSAKCY
9.		GGCGTTACGTCGTCGTCTTTTGCCTCGGACAGAGAACTTGTCCCCGCTAC
10.		ACCCAAATTTGTCACCGCTACTTGCTGGCCAAGGTGCTCTGT
11.		GGTTGGTGACACCCAAGACAGCCAAGTCCAATTCACACCGAGCAGGTCGT
12.		CCAMACGCAAAAACCACACACTCCTTCCAATCCG
13.		GTGKTTGGTGWYKTKGRTGRTKMGTKMCWRSGSAYGRMGGGTRWGSSA
14.		CGAGAGCAGTACAGCGCAGTGGGCAGCACACCCTGAGCAAATGCCTGACC
15.		ACCCTCCAACGAGCGTCGCTGGTGAATTGCGGGGCCTCAAG
16.		YGCSTTYSGGYGWKRTGCCKYTACRWKRWGTGCCRRARCC
17.		GAGGGGTTGTGGGTCTGGGGCATGTCCTCTTATTGGATTCCGGTGAGGTG
18.		ATGTAGCGAGAGAAATTGAGTGAGAAGGCGTGTTGGCTTTGCGTCGCGGC
19.		GGACAAGGCTTCCATTCCTTTACAGCAAGTGCACGTCCCGCTGCTGGAGA
20.		CAATATTGTTAGTCYCTTATTCCTCGGAGGAATGGAATGTCACGGTDTCG
21.		ACGGGGTGCGTCATCACCTCCCATGAAGTCAGAAAACGTTCG
22.		GCGGTTACTATCCTGAGTTGTCGACTTTTTCTGTCTGTTTTTTTTGTTTT
23.		GGCTCTCAGGAACAAGAAGCGACTGTTTCTTTACGCGATACGGGTTTTGA
24.		AGCCTACGGTCTTTCCGTGGCCATGGATG
25.		CGCATCCGCGTTGGCAGAAATTAGTGCAC
26.		TGTACCAGTTGAATGCTGTACCTGGCCGC
27.		CCCCCTGAGTATTGACATTTGGGTTGATAATACATTCCTGTAAGGCGCGG
28.		TGTTGCCACTGTCCGCACTTGGTTTAGTAAGTGGGTATTGCTTTCTTGCC
29.		GCTCAACATACCTGTCACTGAGTGGTGAGGAGCGTGAGCAAATTGATGTA

Supplementary Figure 6. Continued.

Name	*p*-value	Motif Locations
Chr8:2880961-2916647	0.00e+0	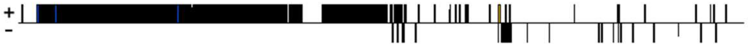

Motif	Symbol	Motif Consensus
1.		GGTGTAAAACACGCATTCTTGCACAACATGCACAACGTTGC
2.		GCGTATTTTCACGTAAAATACGCGTTCATACGTGTGATTGTGCAAAAACA
3.		GGTTTAAAACACGCATTCTTGCACAACATGCACAACGTTGC
4.		GCGTATTTTCACGTAAAATACGCGTTCATACGTGTGATTGTGCAAAAACA
5.		GGTKTAAAACACGCATTCTTGCACAACATGCACAACGTTGC
6.		GCGTATTTTCACGTAAAATACGCGTTCATACGTGTGATTGTGCAAAAACA
7.		GGTTTAAAACACGCATTCTTGCACAACATGCACAACGTTGC
8.		GCGTATTTTCACGTAAAATACGCGTTCATACGTGTGATTGTGCAAAAACA
9.		GGTTTAAAACACGCATTCTTGCACAACATGCACAACGTTGC
10.		GCGTATTTTCACGTAAAATACGCGTTCATACGTGTGATTGTGCAAAAACA
11.		GGTTTAAAACACGCATTCTTGCACAACATGCACAACGTTGC
12.		GCGTATTTTCACGTAAAATACGCGTTCATACGTGTGATTGTGCAAAAACA
13.		GGTTTAAAACACGCATTCTTGCACAACATGCACAACGTTGC
14.		KKTTTAAAACACGCATTCTTGCACAACATGCACAACGTTGC
15.		GTGCACTATTGCGTATTTTCACGTAAAATACGCGTTCATACGTGTGATTG
16.		GGTTTAAAACACGCATTCTTGCACAACATGCACAACGTTGC
17.		GTGCACTATTGCGTATTTTCACGTAAAATACGCGTTCATACGTGTGATTG
18.		GTGCACTATTGCGTATTTTCACGTAAAATACGCGTTCATACGTGTGATTG
19.		GGTTTAAAACACGCATTCTTGCACAACATGCACAACGTTGC
20.		GTGCACTATTGCGTATTTTCACGTAAAATACGCGTTCATACGTGTGATTG
21.		GCATTCTTGCACAACATGCACAACGTTGC
22.		GTGCACTATTGCGTATTTTCACGTAAAATACGCGTTCATACGTGTGATTG
23.		GCATTCTTGCACAACATGCACAACGTTGC
24.		GTGCACTATTGCGTATTTTCACGTAAAATACGCRTTCATRCGTRTGATTG
25.		GCATTCTTGCACAACCTGCACAATGTTGC
26.		GTGCACTATTGCGTATTTTCACGTCAAATACGCATTCATGCGTATGATTG
27.		GCATTCTTGCACAACCTGCACAATGTTGC
28.		GTGCACTATTGCGTATTTTCACGTCAAATACGCATTCATGCGTATGATTG
29.		GCATTCTTGCACAACCTGCACAATGTTGC
30.		GTGCACTATTGCGTATTTTCACGTCAAATACGCATTCATGCGTATGATTG
31.		GCATTCTTGCACAACCTGCACAATGTTGC
32.		GTGCACTATTGCGTATTTTCACGTCAAATACGCATTCATGCGTATGATTG
33.		GCATTCTTGCACAACCTGCACAATGTTGC
34.		GTGCACTATTGCGTATTTTCACGTCAAATACGCATTCATGCGTATGATTG
35.		GCATTCTTGCACAACCTGCACAATGTTGC
36.		GCATTCTTGCACAACCTGCACAATGTTGC
37.		TACGCRTTCATRCGTRTGATTGTGCAAAAACAGTGTTGCAATGCAACATG
38.		CACAAATTGTGCACTATTGCGTATTTTCACGTCAAATACGCATTCATGCG
39.		CGCATTCATGCGTATGATTGTGCAAAAACAGTGTTGCAATG
40.		TGATTGTGCAAAAACAGTGTTGCAATGCAACATGTAAGGTGTTTTGGT
41.		TGATTGTGCAAAAACAGTGTTGCAATGCAACATGTAAGGTGTTTTGGTGT
42.		GTGCAYTATTGCGTATTTTCACGTMAAATACGCRTTCATRC
43.		TGCAAAAACAGTGTTGCAATGCAACATGTAACGTGTTTTGG
44.		TGCAAAAACAGTGTTGCAATGCAACATGTAASGTGTTTTGG
45.		TGCAAAAACAGTGTTGCAATGCAACATGTAAGGTGTTTTGG
46.		TGCAAAAACAGTGTTGCAATGCAACATGTAAGGTGTTTTGG
47.		TGCAAAAACAGTGTTGCAATGCAACATGTAAGGTGTTTTGG
48.		TGCAAAAACAGTGTTGCAATGCAACATGTAAGGTGTTTTGG
49.		TGTTTGTWCACAAAWTGTGCACTATTGCGTATTTTCACGT
50.		TGTTTTGGTKTAAAACACGCATTCTTGCACAACMTGCA
51.		TGTTTGTTCACAAATTGTGCACTATTGCGTATTTTCACGTM
52.		AAACACGCATTCTTGCACAACATGCACAACGTTGCATGTTTGTACACAAA
53.		AACAGTGTTGCAATGCAACATGTAACGTG
54.		AACAGTGTTGCAATGCAACATGTAACGTG
55.		AACAGTGTTGCAATGCAACATGTAACGTG
56.		GTGTTGCAATGCAACATGTGACGTGTTTT
57.		GTGTTGCAATGCAACATGTGACGTGTTTT
58.		GTGTTGCAATGCAACATGTGACGTGTTTT
59.		GTGTTGCAATGCAACATGTGACGTGTTTT
60.		GTGTTGCAATGCAACATGTAACGTGTTTT
61.		TGCAAAAACAGTGTTGCAATGCARCATGT
62.		TKGCGTATTTTCACGTMAAATACGCGTTC
63.		AACATGTAAGGTGTTTTGGTGTAAAACAC
64.		CAATGCAACATGTAACGTGTT
65.		GTTTGTACACAAAATGTGCAC
66.		GTTTGTACACAAAATGTGCAC
67.		GTTTGTACACAAAATGTGCAC
68.		GTTTGTACACAAAATGTGCAC
69.		GTTTGTACACAAAATGTGCAC
70.		GGACTHYWCCTGCGKCCGGYKCYKTTC
71.		TTTGTWCACAAAWTGTGCACT
72.		CACACASVCACACACACMYWCMRCCDCAM
73.		TGMACGGWATRMAGSWRRWGBGSSSWSWARCCMSCCAMCSCRGMSWCTSC
74.		CKMGCTCCACACTCGGWAATAAGGRGGGRAGTDGBAGRRRGGAG
75.		GCAAAAACAGTGTTG
76.		GAGVCAGAGGGGGTDYGCAGTCWHCAHGCAHCMGARAVAGYRWGWRSGRA
77.		GGGAGAMKVTGCCACAGYCGCVAGASCGMTAGCATCTCAGGRCTCWHSG
78.		ATGTTTGTACACAAA
79.		ATGTTTGTACACAAA
80.		ATGTTTGTTCACAAA
81.		ATGTTTGTACACAAA
82.		ATGTTTGTTCACAAA
83.		ATGTTTGTACACAAA
84.		ATGTTTGTTCACAAA
85.		ATGTTTGTACACAAA
86.		ATGTTTGTTCACAAA
87.		ATGTTTGTACACAAA
88.		TGTTTGTTCACAAAT
89.		AGAAGRWGHCAWTTNGCNATCCTTTYKTTYHGDTTTTTGTTATWYGTTTW
90.		GCGCBWNCCTTTTGSNGAASCATACADCC
91.		GWAGAAAMSTYRMYGCWYCYGMWSMGMTGTYCHSKTTTGAAGGARTYA
92.		AASGTGTTTTGGTGT
93.		CASCCYAMSMYMCTCTCYAYAATTTTTSYTSTMSYTC
94.		ACGTGTGATTG
95.		CTTCTTTTGATGTAGGGTGAGGCCAGTTTT
96.		AAACACGSATTCT
97.		AGTGAGTTGTATGACTGTTACCTTAGGTG
98.		CACTGSBRCCRMCATCAGCCATCHC
99.		GCAYVGGVCGVGYVACGRDGNCYCDCBSCTGGGTGCAVACSCYMNS
100.		CTGRYGAHNCTNRCGABGTCMAMABGGAG

Supplementary Figure 6. Continued.

Name	*p*-value	Motif Locations
Chr8:2880961-2916647	0.00e+0	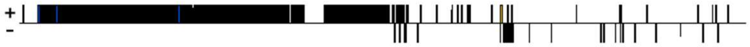

Motif	Symbol	Motif Consensus
1.		GGTGTAAAACACGCATTCTTGCACAACATGCACAACGTTGC
2.		GCGTATTTTCACGTAAAATACGCGTTCATACGTGTGATTGTGCAAAAACA
3.		GGTTTAAAACACGCATTCTTGCACAACATGCACAACGTTGC
4.		GCGTATTTTCACGTAAAATACGCGTTCATACGTGTGATTGTGCAAAAACA
5.		GGTKTAAAACACGCATTCTTGCACAACATGCACAACGTTGC
6.		GCGTATTTTCACGTAAAATACGCGTTCATACGTGTGATTGTGCAAAAACA
7.		GGTTTAAAACACGCATTCTTGCACAACATGCACAACGTTGC
8.		GCGTATTTTCACGTAAAATACGCGTTCATACGTGTGATTGTGCAAAAACA
9.		GGTTTAAAACACGCATTCTTGCACAACATGCACAACGTTGC
10.		GCGTATTTTCACGTAAAATACGCGTTCATACGTGTGATTGTGCAAAAACA
11.		GGTTTAAAACACGCATTCTTGCACAACATGCACAACGTTGC
12.		GCGTATTTTCACGTAAAATACGCGTTCATACGTGTGATTGTGCAAAAACA
13.		GGTTTAAAACACGCATTCTTGCACAACATGCACAACGTTGC
14.		KKTTTAAAACACGCATTCTTGCACAACATGCACAACGTTGC
15.		GTGCACTATTGCGTATTTTCACGTAAAATACGCGTTCATACGTGTGATTG
16.		GGTTTAAAACACGCATTCTTGCACAACATGCACAACGTTGC
17.		GTGCACTATTGCGTATTTTCACGTAAAATACGCGTTCATACGTGTGATTG
18.		GTGCACTATTGCGTATTTTCACGTAAAATACGCGTTCATACGTGTGATTG
19.		GGTTTAAAACACGCATTCTTGCACAACATGCACAACGTTGC
20.		GTGCACTATTGCGTATTTTCACGTAAAATACGCGTTCATACGTGTGATTG
21.		GCATTCTTGCACAACATGCACAACGTTGC
22.		GTGCACTATTGCGTATTTTCACGTAAAATACGCGTTCATACGTGTGATTG
23.		GCATTCTTGCACAACATGCACAACGTTGC
24.		GTGCACTATTGCGTATTTTCACGTAAAATACGCRTTCATRCGTRTGATTG
25.		GCATTCTTGCACAACCTGCACAATGTTGC
26.		GTGCACTATTGCGTATTTTCACGTCAAATACGCATTCATGCGTATGATTG
27.		GCATTCTTGCACAACCTGCACAATGTTGC
28.		GTGCACTATTGCGTATTTTCACGTCAAATACGCATTCATGCGTATGATTG
29.		GCATTCTTGCACAACCTGCACAATGTTGC
30.		GTGCACTATTGCGTATTTTCACGTCAAATACGCATTCATGCGTATGATTG
31.		GCATTCTTGCACAACCTGCACAATGTTGC
32.		GTGCACTATTGCGTATTTTCACGTCAAATACGCATTCATGCGTATGATTG
33.		GCATTCTTGCACAACCTGCACAATGTTGC
34.		GTGCACTATTGCGTATTTTCACGTCAAATACGCATTCATGCGTATGATTG
35.		GCATTCTTGCACAACCTGCACAATGTTGC
36.		GCATTCTTGCACAACCTGCACAATGTTGC
37.		TACGCRTTCATRCGTRTGATTGTGCAAAAACAGTGTTGCAATGCAACATG
38.		CACAAATTGTGCACTATTGCGTATTTTCACGTCAAATACGCATTCATGCG
39.		CGCATTCATGCGTATGATTGTGCAAAAACAGTGTTGCAATG
40.		TGATTGTGCAAAAACAGTGTTGCAATGCAACATGTAAGGTGTTTTGGT
41.		TGATTGTGCAAAAACAGTGTTGCAATGCAACATGTAAGGTGTTTTGGTGT
42.		GTGCAYTATTGCGTATTTTCACGTMAAATACGCRTTCATRC
43.		TGCAAAAACAGTGTTGCAATGCAACATGTAACGTGTTTTGG
44.		TGCAAAAACAGTGTTGCAATGCAACATGTAASGTGTTTTGG
45.		TGCAAAAACAGTGTTGCAATGCAACATGTAAGGTGTTTTGG
46.		TGCAAAAACAGTGTTGCAATGCAACATGTAAGGTGTTTTGG
47.		TGCAAAAACAGTGTTGCAATGCAACATGTAAGGTGTTTTGG
48.		TGCAAAAACAGTGTTGCAATGCAACATGTAAGGTGTTTTGG
49.		TGTTTGTWCACAAAWTGTGCACTATTGCGTATTTTCACGT
50.		TGTTTTGGTKTAAAACACGCATTCTTGCACAACMTGCA
51.		TGTTTGTTCACAAATTGTGCACTATTGCGTATTTTCACGTM
52.		AAACACGCATTCTTGCACAACATGCACAACGTTGCATGTTTGTACACAAA
53.		AACAGTGTTGCAATGCAACATGTAACGTG
54.		AACAGTGTTGCAATGCAACATGTAACGTG
55.		AACAGTGTTGCAATGCAACATGTAACGTG
56.		GTGTTGCAATGCAACATGTGACGTGTTTT
57.		GTGTTGCAATGCAACATGTGACGTGTTTT
58.		GTGTTGCAATGCAACATGTGACGTGTTTT
59.		GTGTTGCAATGCAACATGTGACGTGTTTT
60.		GTGTTGCAATGCAACATGTAACGTGTTTT
61.		TGCAAAAACAGTGTTGCAATGCARCATGT
62.		TKGCGTATTTTCACGTMAAATACGCGTTC
63.		AACATGTAAGGTGTTTTGGTGTAAAACAC
64.		CAATGCAACATGTAACGTGTT
65.		GTTTGTACACAAAATGTGCAC
66.		GTTTGTACACAAAATGTGCAC
67.		GTTTGTACACAAAATGTGCAC
68.		GTTTGTACACAAAATGTGCAC
69.		GTTTGTACACAAAATGTGCAC
70.		GGACTHYWCCTGCGKCCGGYKCYKTTC
71.		TTTGTWCACAAAWTGTGCACT
72.		CACACASVCACACACACMYWCMRCCDCAM
73.		TGMACGGWATRMAGSWRRWGBGSSSWSWARCCMSCCAMCSCRGMSWCTSC
74.		CKMGCTCCACACTCGGWAATAAGGRGGGRAGTDGBAGRRRGGAG
75.		GCAAAAACAGTGTTG
76.		GAGVCAGAGGGGGTDYGCAGTCWHCAHGCAHCMGARAVAGYRWGWRSGRA
77.		GGGAGAMKVTGCCACAGYCGCVAGASCGMTAGCATCTCAGGRCTCWHSG
78.		ATGTTTGTACACAAA
79.		ATGTTTGTACACAAA
80.		ATGTTTGTTCACAAA
81.		ATGTTTGTACACAAA
82.		ATGTTTGTTCACAAA
83.		ATGTTTGTACACAAA
84.		ATGTTTGTTCACAAA
85.		ATGTTTGTACACAAA
86.		ATGTTTGTTCACAAA
87.		ATGTTTGTACACAAA
88.		TGTTTGTTCACAAAT
89.		AGAAGRWGHCAWTTNGCNATCCTTTYKTTYHGDTTTTTGTTATWYGTTTW
90.		GCGCBWNCCTTTTGSNGAASCATACADCC
91.		GWAGAAAMSTYRMYGCWYCYGMWSMGMTGTYCHSKTTTGAAGGARTYA
92.		AASGTGTTTTGGTGT
93.		CASCCYAMSMYMCTCTCYAYAATTTTTSYTSTMSYTC
94.		ACGTGTGATTG
95.		CTTCTTTTGATGTAGGGTGAGGCCAGTTTT
96.		AAACACGSATTCT
97.		AGTGAGTTGTATGACTGTTACCTTAGGTG
98.		CACTGSBRCCRMCATCAGCCATCHC
99.		GCAYVGGVCGVGYVACGRDGNCYCDCBSCTGGGTGCAVACSCYMNS
100.		CTGRYGAHNCTNRCGABGTCMAMABGGAG

Supplementary Figure 6. Continued.

Name	*p*-value	Motif Locations
Chr8:1067281-1108401	0.00e+0	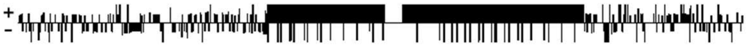

Motif	Symbol	Motif Consensus
1.		CCTCTGAGTCCGGAGKGGGKGGGGGGGGGKKGKKGGSGG
2.		CCCCGGGTTCCAGCGCCATCACTGATCGCCGTACTAACGAGGCCTGTGGC
3.		CCCCGGGTTCCAGCGCCATCACTGATCGCCGTACTAACGAGGCCTGTGGC
4.		CCCCGGGTTCCAGCGCCATCACTGATCGCCGTACTAACGAGGCCTGTGGC
5.		CCCCGGGTTCCAGCGCCATCACTGATCGCCGTACTAACGAGGCCTGTGGC
6.		GGGGGGGGGGGTTGGGGGCGGATGAAACAGCCGTTCCGATGCACATCTA
7.		CCTCTGAGTCCGGAGTGGGTKGGGGGGGGGGKTKGGGGSSG
8.		CGGACGGGATATGGTGCATTCGGCCAAGTATGGTCGTACCCGACGTCCGG
9.		CGGACGGGATATGGTGCATTCGGCCAAGTATGGTCGTACCCGACGTCCGG
10.		CGGACGGGATATGGTGCATTCGGCCAAGTATGGTCGTACCCGACGTCCGG
11.		CGGACGGGATATGGTGCATTCGGCCAAGTATGGTCGTACCCGACGTCCGG
12.		GGGGGGGGGTTGGGGGCGGATGAAACAGCCGTTCCGATGCA
13.		CCTTCCCCATTTTTCCCTCCTTCCTTGCTGTCTTGTTGTCAGCCCTGTGC
14.		GCGTGTGTGTGTTTGTGCTGGAGCCGAAGGGAGAGAAGGGG
15.		CCTTCCCCATTTTTCCCTCCTTCCTTGCTGTCT
16.		CCTTCCCCATTTTTCCCTCCTTCCTTGCTGTC
17.		GAAGGGAGAGAAGGGGAACTTTTAACGGCAACCAGACAG
18.		GTGCTGGAGCCGAAGGGAGAGAAGGGGAACTTTTAACGGCAACCAGACAG
19.		CCTTCCCCATTTTTCCCTCCTTCCTTGCTGTC
20.		CCACTCCATACCATCCCCCCTCCAGCACC
21.		TCTGTAGGTGCTGGAGGGGGGATGGTATGGAG
22.		CCACTCCATACCATCCCCCCTCCAGCACC
23.		AAAAAGTTTCTACCACCATGGCCGATCTC
24.		AAAAAGTTTCTACCACCATGGCCGATCTC
25.		AGACACCATTTCCATCACACTACACCACA
26.		AGACACCATTTCCATCACACTACACCACA
27.		GCGTGTGTGTGTTTGTGCTGGAGCCGAAGGGAGAGAAGGGG
28.		GCACCTTTCTTACCACTCCATACCATCCCCCCTCCAGCACCTACAGACAC
29.		GAGATCGGCCATGGTGGTAGMAACTTTTTCCGGGAGTATGG
30.		CCGATACCCGTCAAACTTGTTCATGAGGGATTTGAAGTGTAGGAGACGCG
31.		CCGATACCCGTCAAACTTGTTCATGAGGGATTTGAAGTGTAGGAGACGCG
32.		YCGATACCCGTCAAACTTGTTCATGAGGGATTTGAAGTGTAGGAGACGCG
33.		CCGATACCCGTCAAACTTGTTCATGAGGGATTTGAAGTGTAGGAGACGCG
34.		GCCCTGTGCGCAAAAATTACCGTTATTGTATATGAGATCGGCCATGGTGG
35.		TCAGTGTGTGTTTCCTCTTGTATTTGCTTGTGTTTATGCGTGTGTGTGTT
36.		GAAACGGACGATTGGCATAGATTTAACACAAGTTGAATGACGGCTGCAGC
37.		GAAACGGACGATTGGCATAGATTTAACACAAGTTGAATGACGGCTGCAGC
38.		GAAACGGACGATTGGCATAGATTTAACACAAGTTGAATGACGGCTGCAGC
39.		CGGCAACCAGACAGTGAAGAAACTTAGAAACGGACGATTGG
40.		CACAAGTTGAATGACGGCTGCAGCTCTAGGAAAAAAGTTTGGGAGAGTAC
41.		CTTTTTCCGGGAGTATGGTCAGTGTGTGTTTCCTCTTGTATTTGCTTGTG
42.		CCACACTAAAAAGTGGAAGAAAACATAACTTTCCTCTGAGTCCGGAGTGG
43.		ACAGCCGTTCCGATGCACATCTATTATTCCATACAAAAGGGTAGGAAAGC
44.		ACAGCCGTTCCGATGCACATCTATTATTCCATACAAAAGGGTAGGAAAGC
45.		CTACACCACACTAAAAAGTGGAAGAAAACATAACTTTCCTCTGAGTCCGG
46.		TCAGTGTGTGTTTCCTCTTGTATTTGCTTGTGTTTATGCGTGTGTGTGTT
47.		CATCTATTATTCCATACAAAAGGGTAGGAAAGCAAGTTATGAAGTTTCCC
48.		CCGGGAGTATGGTCAGTGTGTGTTTCCTCTTGTATTTGCTT
49.		CCATACAAAAGGGTAGGAAAGCAAGTTATGAAGTTTCCCTG
50.		TGTTGTCAGCCCTGTGCGCAAAAATTACCGTTATTGTATA
51.		TGTTGTCAGCCCTGTGCGCAAAAATTACCGTTATTGTATAT
52.		AACTTTTAACGGCAACCAGACAGTGAAGA
53.		TCTAGGAAAAAAGTTTGGGAGAGTACAAC
54.		TCTAGGAAAAAAGTTTGGGAGAGTACAAC
55.		TCTAGGAAAAAAGTTTGGGAGAGTACAAC
56.		AAAAAAAGAAAAGAAGAAAAGCACCTTTC
57.		AAGAAAAGAAGAAAAGCACCTTTCTTACC
58.		AAAAAAAGAAAAGAAGAAAAGCACCTTTC
59.		CAKCAMKGAWSGMSGKAMWAAYGRGGMMKGWGRCMGSKWAACTTCAYA
60.		TAACACAAGTTGAATGACGGCTGCAGCTCTAGGAAAAAAGTTTGGGAGAG
61.		AAGTTATGAAGTTTCCCTGCC
62.		TAAACCGAATGCGTGCATTTA
63.		TAAACCGAATGCGTGCATTTA
64.		TAAACCGAATGCGTGCATTTA
65.		TAAACCGAATGCGTGCATTTA
66.		GCAAAAATTACCGTTATTGTA
67.		CTAAAAAGTGGAAGAAAACAT
68.		CMRTTCCGATRCACATCTATTATTCCATACAAAAGGGTAGGAAAGCAAGT
69.		AAAAAAAAAAAAAGAAAAGAA
70.		CTAAAAAGTGGAAGAAAACAT
71.		AAGTTATGAAGTTTCCCTGCT
72.		GTGCTGGAGCCGRAGGKAGAGARGGGGAA
73.		CACCATTTCCATCACACTACA
74.		GTTGTCAGCCCTGTGCGCAAAAATTACCG
75.		GTGTGTTTCCTCTTGTATTTGCTTGTGTT
76.		CTTTTAACGGCAACCAGACAGTGARGAMA
77.		YTWBYVCBGATGATACKGBKBAYDGVACSCTGGCACCHGTYBAGGKWBC
78.		CGCTTAACTTCACAA
79.		CGCTTAACTTCACAA
80.		CGCTTAACTTCACAA
81.		CGCTTAACTTCACAA
82.		AAAGCAGBGTTSCHCGHBMARGGAACGCTGCWGGCTCTTGG
83.		TATGRGAKCGGCCAKGGTGGT
84.		CGGACGGGATATGGTGCATTCGGCCAAGTATGGTCGTACCCGACGTCCGG
85.		GAAAVTCASKGHCCMHACAARAYRMTVCTGMTCCCTGYGCTGTWKGAKG
86.		ACGGACGATTGGCATAGATT
87.		CCGATACCCGTCAAACTTGTTCATGAGGGATTTGAAGTGTAGGAGACGCG
88.		CTTTTTTTTTTTTTTTNWT
89.		CCGGGAGTATG
90.		GDGGVKGDGGBDVGTTKGGRGCGGATGAA
91.		TGTAGTGTGATGGAA
92.		TCCCTNKTCTTRTCVBGCAGG
93.		TGYGTGTGTGTGTTT
94.		CCCCGGGTTCCAGCG
95.		CACCATTTCCATCAC
96.		TGTGCTGGAGC
97.		TGAAGAAACTT
98.		TGAAGAAACTT
99.		GVAAAHACAAMAGCBNATGNDCCTYVCGHGGCAATTGHWC
100.		CCACACTAAAAAGTGGAAGAAAACATAAC

Supplementary Figure 6. Continued.

Name	*p*-value	Motif Locations
Chr9:2307676-2375562	0.00e+0	+ - 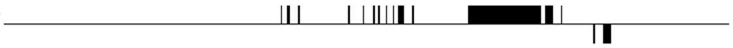

Motif	Symbol	Motif Consensus
1.		CCGCCGGCCAGCCACAGAGCCGAAAGAAGCCGGSCTGCGCG
2.		TCGGCAGAGGCGCCCCTGGCTCCTCCCCATCACCCCCTGCC
3.		AGAGAGCGCTGGTAAAAGACGGCGGGTCGCCGCCCACATGGTGGTTGCCG
4.		CCCTCYAAAATCTGGCCGCCGCCGGCTGGTCCTGGCGCGCCCCAGAAACG
5.		GGCGGTCGGCATGCCCCTCTACATGCCTACTGAGCAGGCGRACGGGCCCC
6.		CCCCGCCCAATCATCACCCGCCGCTAATAAACGGCGGCAGC
7.		GCCGCATACTGCGGGAATCTGGAAGGTGGGGTCGGATGACC
8.		TAAGCGCGTTGGGGTTCTYGATGGGCGCTTTCATGGCTTATACGTGCTCG
9.		GCAGCGGAAAGAGCTCCCCCGTCGGCCCCCAACCAAGAGGAGCCACGGCG
10.		TGGTATGAGAAGCTCCCAGTAGCAGCTGGGCCAACACACGC
11.		ACCCCATGTAGTATATATGCGGCCCCGCTTATTCCGCCCCC
12.		CGTAGCGCATTTGAAAGGGCTCGACCAGCCCAATGCATGAG
13.		CGTAGCGCATTTGAAAGGGCTCGACCAGCCCAATGCATGAG
14.		GGCATGGCAACACCAAATATCCCCTTTCAGGGTTTCGCCTCATTTTGCCG
15.		CACTCAGCTCAACCGTCCTTCTTCTTTGCGTTGTTGTTGTCGCCGTTGTG
16.		TYTTTATTTGCKAYGCTGACACACACACKCAMACACRCWCACKCACWC
17.		ACACATCACTTTCTTACACATATAGGCGCTTTAAAGTCTGCTGCCCGCCG
18.		TGTTCTTAACCTGGTTATACCCGCAATATGCCAGCTGCACGTTCAGTTCG
19.		TCTGGCACGACAGTAAAATATGGCAAGTGTCTCAAAACTGCCTGTACAGC
20.		TCCCCCATAACGCCTTAAGCACAAGACCC
21.		AACCGTGGTCAAAWCGACATGACGACCAC
22.		TCTCCCGTTCATTTTTACGCAGTCGGACAATTTCATGTCGC
23.		GGTTGATTGCYTTAATGTTCTGTTGCAGATGGCGATTCACC
24.		CGGTTACTGCGACAAAGAGGTCAYAARAAAAWTAGGKGCDG
25.		TGGGACACASCCATGCTTTCAACTAACGCTATTATTAGAAC
26.		AACCGTGGTCAAATCGACATGACGACCAC
27.		TAGAACAGTTTCTGTACTATATTGGTATGAGAAGCTCCCAG
28.		AACAACGGTTACTGCGACAAAGAGGTCAC
29.		TATATGCAAATATAATTATACTATAGCTTATGGTTTTCTTGCATATCTGT
30.		TATATATTTCTTTATTTATTTATTTGTTTGTTTATATCTATATCTATATC
31.		TCGAACCTAATATTTTTTCAAAAAATGGAAATTTTGAAAAA
32.		GTTATATACTTTTCCAATTTTGGGGCCGG
33.		CCCCTATTCATGTTATTAGCCGCCATTAA

Name	*p*-value	Motif Locations
Chr10:4041817-4121137	0.00e+0	+ - 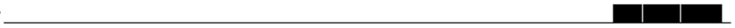

Motif	Symbol	Motif Consensus
1.		TACTGCTCTYAKTATMATATTCGTATATACACACTCACAACACTCTC
2.		TATAYACACACTCACAACACTCTCCTATTATYAYTATT
3.		TACTGCTCTTATTATAATATTYGTATATACACACWCWCAMMACWCTCYY
4.		TACTGCTCTTATTATAATATTYGTATATACACACTCACAACACTCTCCTA
5.		TACTGCTCTTATTATAATATTYGTATATACACACTCACAACACTCTCCTA
6.		TATACACACWCTCACAACACTCTCCTATTATTATYAT
7.		ATATACACACACTCACAACACTCTCCTATTATYATYATT
8.		TATACACACWCTCACAACACTCTCCTATTATTATYATT
9.		TACTGCTCTTATTATAATATTHRTATAYACACACTCACAACACWCTC
10.		TACTRCTCTYATTATAATATTCGTATATACACACTCACAACACTCTCCTA
11.		TATACACACACTCACAACACTCTCCTATTATTATYWT
12.		TACTGCTCTHATTATAATATTCGTATATACACACACTCACAACACTCTCM
13.		TACTGCTCTYATTATAATATTYGTATATACACACWCWCAMMACWCTCY
14.		TDTATRYACACACTCACAACACTCTCCTATTATTATYAT
15.		TACTGCTCTYATTATAATATTCGTATATACACACWCWCAMMACWCTC
16.		TACTGCTCTTATTATAATATTYGTATATACACACWCTCACAACACTCTYC
17.		TACTGCTCTTATTATAATATTCDTATAYACACACTCACAACACWCTC
18.		TCGTATATACACACTCACAACACTCTCCTATTATTAYTATT
19.		TATACACACACTCACAACACTCTYCTATTATHATYAT
20.		TACTRCTCTTATTATAATATTCGTATATACACACWYWCAMMACWCTCY
21.		CTGCTYTYWTTATAATATTCGTATATACACACWCWCAMMACWCTMYYMT
22.		TCGTATATACACACTCACAACACTCTCCTATTATTAT
23.		TATACACACWCTCACAACACTCTCCTATTATTATYATC
24.		TATACACACWCTCACAACACTCTYCTATTATHWTTAT
25.		ATATACACACACTCACAACACTCTCCTATTATYATTATT
26.		TACTGCTCTYAKTATMATATTCGTATATACACACWCWCAMMACWCTCWYM
27.		TACTRCTCTYATYATAATATTCGTATATACACACWCWCAMMACWCWY

Supplementary Figure 6. Continued.

Name	*p*-value	Motif Locations
Chr11:4662356-4674634	0.00e+0	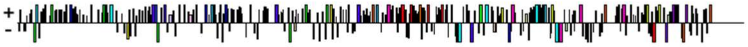

Motif	Symbol	Motif Consensus
1.		TDSRRNHCGACRBCTGCCBHCBBSTKGCVGCCDCSCGCC
2.		TTARAAAGTAACNHRHAGGNYGGCAGBVCNCTVCATVGRBKATGGGSGTG
3.		CGGCNAARAAGTGC
4.		TCSSASTGCCAMSMGKWGCC
5.		CCAACKGCGTYGCGGAAA
6.		GGCAMCCCGTCMCTG
7.		GAGCGTSTCCWTCGG
8.		CCVHSSATDCACANAWNGRAACAAACAWCAAMWAAANAAVNCWTC
9.		AACMHGBHDAAHHSTCGTBTTTGTBATTGCTGGT
10.		RAATGCDRBRAGADATGCTGCYGRSGT
11.		CCGAACSWRSKYCTMRTCSCC
12.		GCRCCMGCCCGATG
13.		CCCCCTCC
14.		GCCGCGCGG
15.		CACRGRYGCCTBCTNCNGT
16.		CCTCTTTCCCCT
17.		CCGGTACSGGTTSTSYSGGT
18.		CCACASCGGCVTCAC
19.		GASCGGAAGCRACCT
20.		TGTGGTCSGTCTCT
21.		CTGGRGGWGCAACCT
22.		TTCCAAGCATDCCCACDSCTHCNMAKTCAAAAAGGGVG
23.		AAGGAGGGAGA
24.		CCGGTTCC
25.		TTTCCVBCTHAACATGGDVTGBGWT
26.		GGAACCGG
27.		CBTGMTCCTYGTGDVAADBGTBTAAADAVBGGAGDC
28.		WAAACARTVATAWCACCACATDTATCACANATTKMWGRNAVAMWDTA
29.		GYACWWKATTCCAGBTKGSA
30.		GGTGTGMGGGA
31.		CCACCAC
32.		GSGGGGCWGTTKCG
33.		CCAACAGC
34.		CCTCTCG
35.		CCYCMSCCSMAAATGCTKGCC
36.		CCACCSCAAC
37.		CACGTYGCTG
38.		CCACSGCAGC
39.		ACGCCTSGCTGT
40.		GGCACSAGCTG
41.		ATCCCWCCCCC
42.		GGCGYCGGC
43.		GTGCCAACAAGAADAWWWAAANGRMGT
44.		ACATAGCCACC
45.		ATGCGGTCTSAGAA
46.		GTGAGAACACA
47.		CACCTTYCTGTG
48.		GGATGCGAT
49.		ACTSASGSACAMAGCGTARCA
50.		GATCACACAC
51.		GCWCATATCYGTGC
52.		AGGSGTSWTRGKGCMATGGTC
53.		GCACACAC
54.		TCGCSTGTSSTCKGGT
55.		GGGGAG
56.		GCGACGAWGTGT
57.		CTGGCAGKSCATG
58.		GGCCGGC
59.		GCGGAWGCAC
60.		CGGCWMKTCGCTGTG
61.		GCCCKCGSAMCCA
62.		CGCCTCTMCSCGA
63.		AAGGRCCCKCAGAAA
64.		GTGTGTTATSCG
65.		AACGATGATSKCGTG
66.		GTTGAAACCGA
67.		CAGATTTYACSGCGA
68.		CCAACCTC
69.		TTGTTSCACAACG
70.		GAGCKTGACACC
71.		GATCCKGTGCTG
72.		AAGGGADATGAAA
73.		CCTCCTGTSMCGAT
74.		TGACAACACTAHABGDGHTRATC
75.		TGCGGWGTRGCACNGAAGAT
76.		CTTCCMTACGG
77.		GCCACTSTTTG
78.		CAGTTCTCC
79.		TTGTGTGGG
80.		CTTCACASTGYCC
81.		AACATGCAAAC
82.		GGGMGCYAWGTTMCRTCGC
83.		ATGSGTRTCCGWRACACSC
84.		TGCGRCAGWMGTCC
85.		GGGGAT
86.		CTCGGCC
87.		CCCGGATRCG
88.		GAAGCAGRCAATT
89.		CTCCCSTRCGG
90.		GCACRCAWCATGSCA
91.		TCGCKTCTCSRTCRCTTT
92.		CCCAACWGCATG
93.		GGGTMKTGACAGAA
94.		AGCTSATGGTGSG
95.		CCGTTYTCC
96.		ASCTCCG
97.		GTTCCTTGT
98.		CCGCSA
99.		TTCTCTCTTGTG
100.		TTGGATGCAAC

Supplementary Figure 7. RAP1 ChIP-seq analysis with various alignment filtering. A-D) Triangle Hi-C heatmap of *T. brucei* SM427 (top) with BDF2 ChIP-seq and RAP1 ChIP-seq (below). RAP1 ChIP-seq was performed with Oxford nanopore sequencing with all mapped reads [all reads], filtered for only primary reads [primary], and filtered with mapQ = 10 [mapQ = 10]. Notice no significant change in RAP1 mapped to subtelomeric regions and boundaries. Four different chromosomes are shown: A) Chromosome 10, B) Chromosome 8, C) Chromosome 9, and D) Chromosome 11. TADs, identified at 10 Kb resolution from *T. brucei* SM427 Hi-C matrix, and compartments are also indicated. Heatmaps were generated from a 10 kb resolution matrix, balanced using the Knight-Ruiz method. Hi-C data is the sum of three biological replicates. ChIP-seq show the average of three biological replicates.

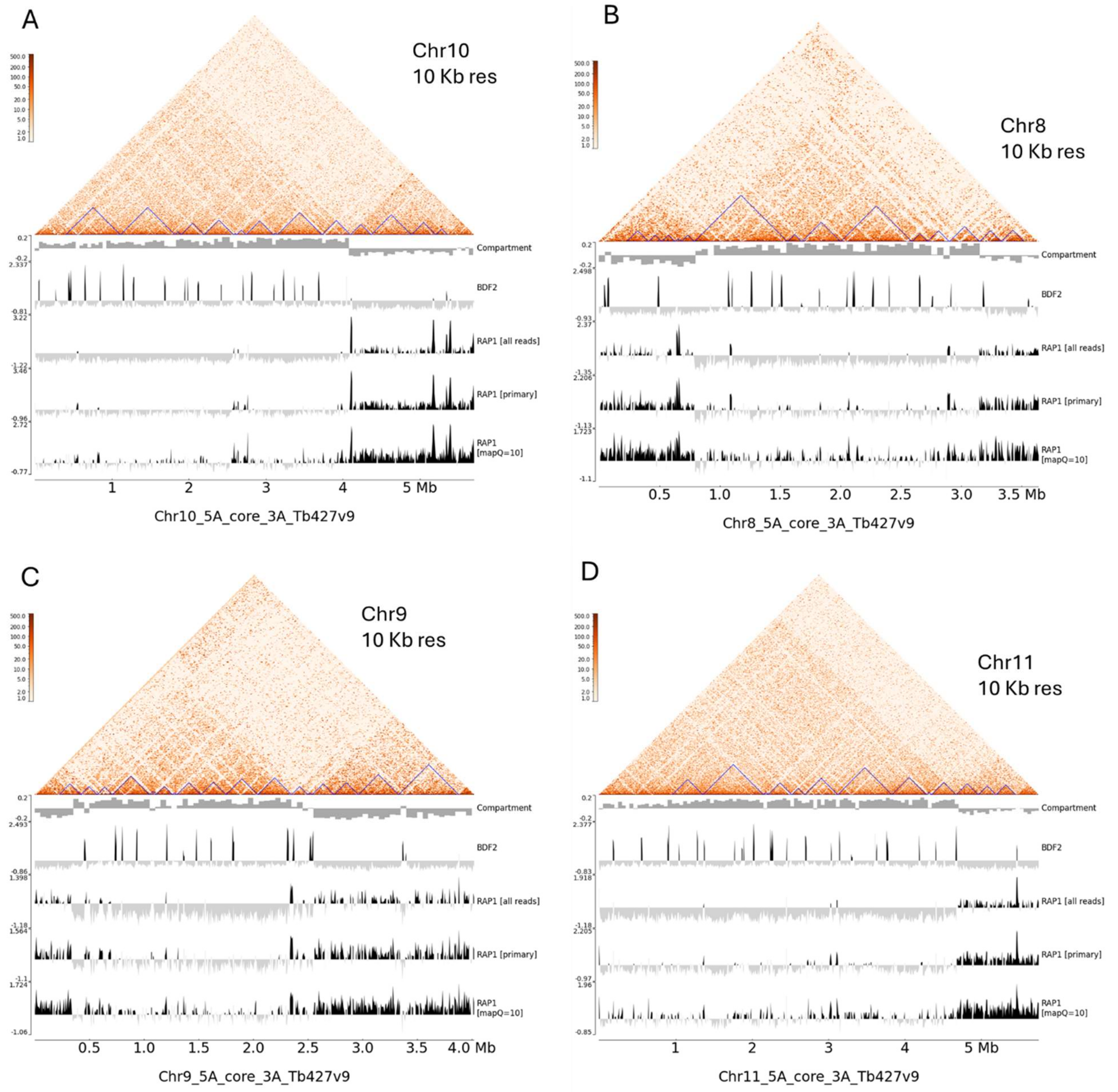

Supplementary Figure 8. Comparison of Hi-C from SM427 and conditional null PIP5Pase. A) Principal component analysis plot of Hi-C summed dataset (3 biological replicates for each group) of *T. brucei* SM427 strain comparing to conditional null PIP5Pase Tet+ (non-knockdown) and Tet – (24h knockdown). PCA sample size of 100,000. B) Hi-C count vs distance plot shows the decay of interactions per genomic distance in bp. The noise at longer distances is expected because contact frequencies at large genomic separations are lower and thus affected by sequencing depth. C) Comparison of compartments between SM427 and conditional null PIP5Pase Tet + (no knockdown) and Tet – (24h knockdown). Data was generated from matrices corrected using the Knight-Ruiz method and normalized to the smallest matrix. The data in A used matrices at 10 Kb resolution, whereas B-C used matrices at 50 Kb resolution.

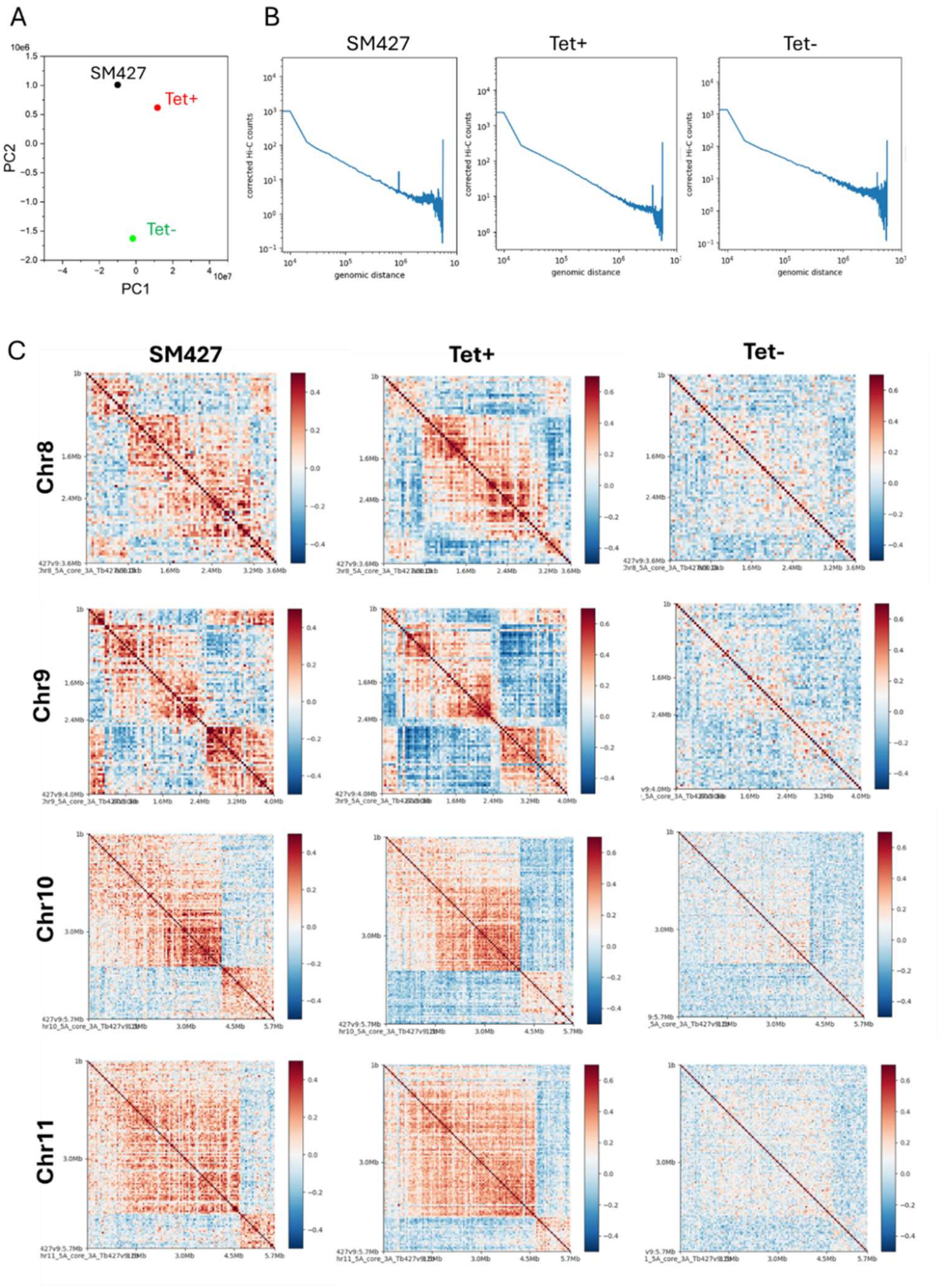

Supplementary Figure 9. Compartment disruption after PIP5Pase knockdown. Comparison of Hi-C matrices between PIP5Pase Tet + (no knockdown) and Tet – (24h knockdown) in the same graph. Heatmaps from matrices at 50 Kb resolution, normalized for the smallest matrix, and corrected using the Knight-Ruiz method. Notice the disruption of compartment contacts after PIP5Pase knockdown. Heatmaps for chromosomes 7, 8, 9, and 11 are shown. Below each graph, compartments identified using FAN-C are shown. Hi-C data is the sum of three biological replicates.

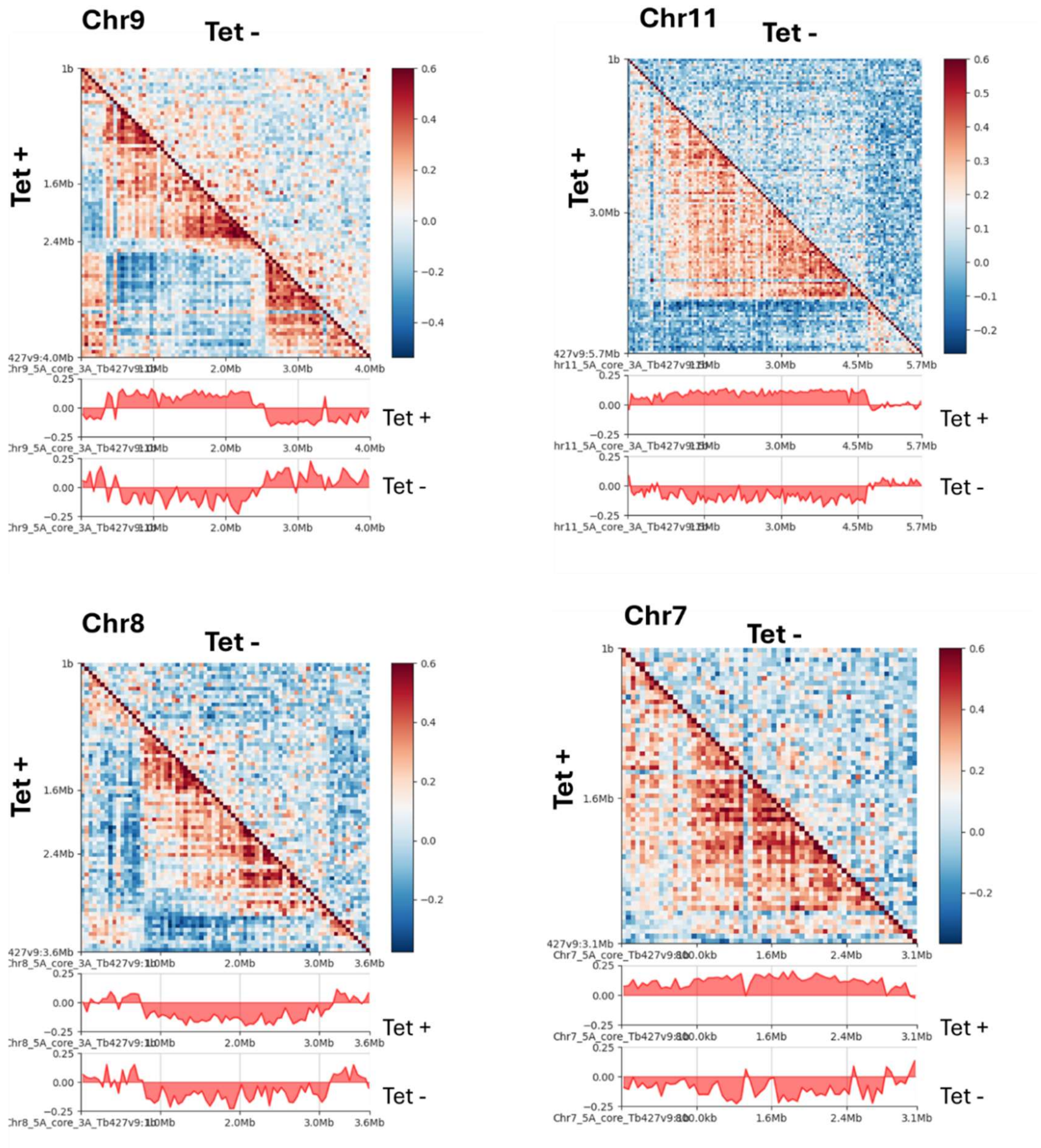

Supplementary Figure 10. PIP5Pase knockdown disrupts chromatin contacts, RAP1 binding to DNA, and VSG gene silencing. Hi-C heatmaps of chromosome 11, 10, 8, and 7 at 10 Kb resolution in cells expressing PIP5Pase (Tet +) or after its 24h knockdown (Tet -). Matrices were normalized to the smallest matrix and corrected using the Knight-Ruiz method. Below the heatmap, it shows compartments, RAP1-HA ChIP-seq in cells exclusively expressing PIP5Pase (WT) or its catalytically inactive mutant (Mut, D362A/N360A), and an RNA-seq heatmap comparing cells exclusively expressing PIP5Pase (WT) with those expressing its catalytically inactive mutant (Mut). RNA-seq and ChIP-seq were performed with Oxford nanopore sequencing. Hi-C data is the sum of three biological replicates for each group. ChIP-seq and RNA-seq results are presented as the average of three biological replicates.

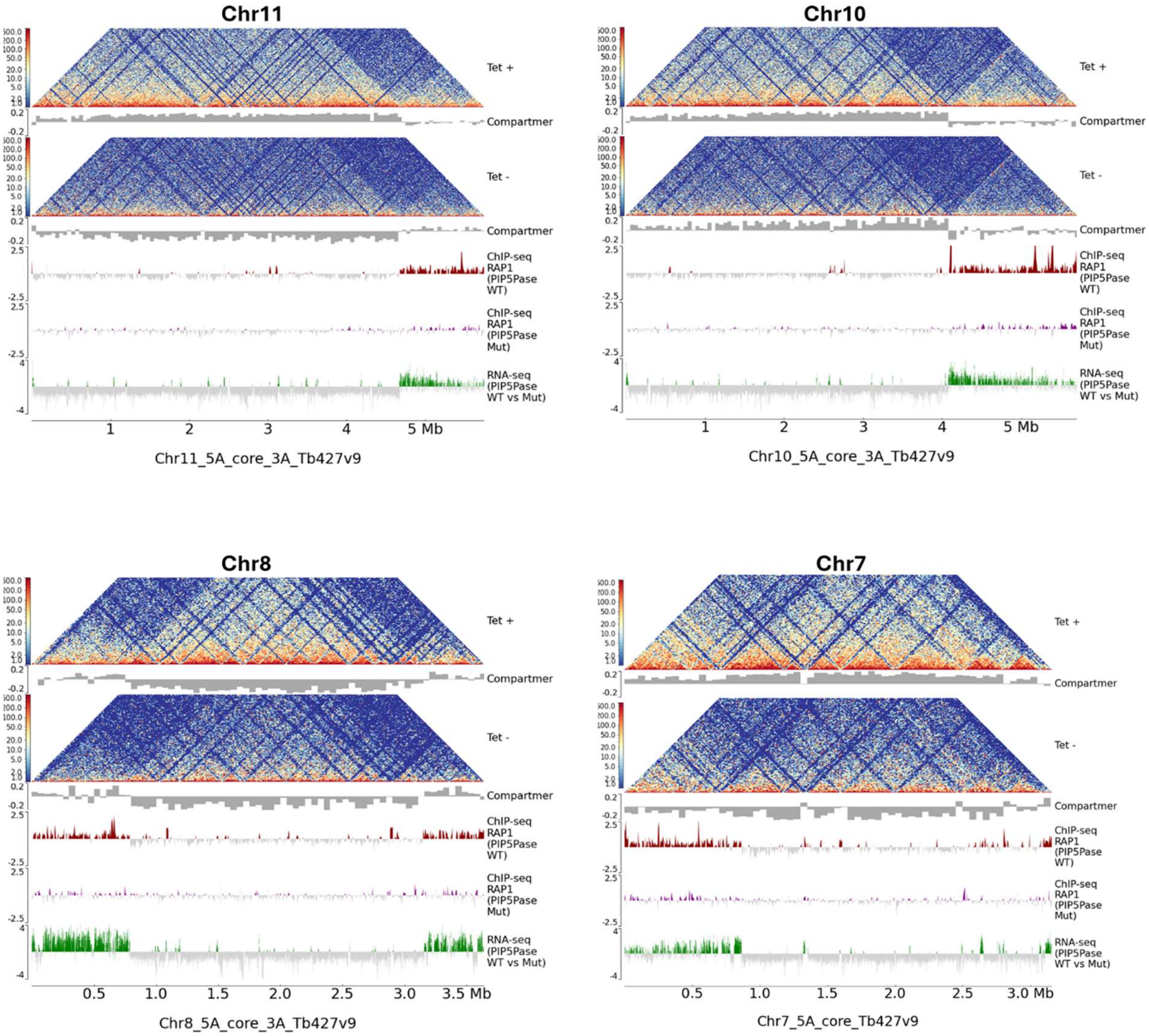

Supplementary Figure 11. Quantification of boundary contacts before and after PIP5Pase knockdown. A) Fold-change comparison between the mean Hi-C contact counts of boundary-boundary interactions and the mean counts of boundary interactions with other chromosome regions. TetP shows cells expressing PIP5Pase, whereas TetM shows cells in which PIP5Pase was knocked down for 24h (TetM). The box indicates interquartile range (25-75%); whiskers with maxima and minima show ± standard deviation of the mean. Center line is the median; central square is the mean. Dots indicate outliers. The mean fold change values are shown above each box. The *p*-value shows a comparison between TetM and TetP using a two-sample two-sided t-test. B-C) Diagram of contact boundaries – determined by Hi-C using a 10 kb resolution matrix normalized to the smallest matrix and corrected using the Knight-Ruiz method– among chromosomes from cells expressing PIP5Pase (TetP) or in which PIP5Pase was knocked down for 24h (TetM). Hi-C data is the sum of three biological replicates for each group. Source data are provided as a Source Data file.

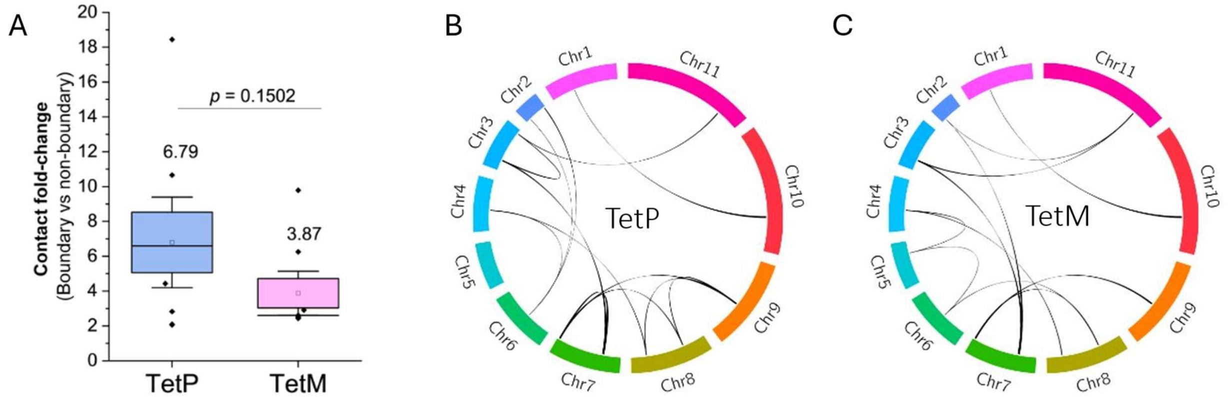

Supplementary Table 1. Statistics of Hi-C sequencing, alignment, and contacts. Sequencing performed by Illumina NovaSeq. R1 and R2 refer to Illumina paired reads. * Sequencing performed with Oxford nanopore sequencer MinION. RP, biological replicates.

**Samples**	**Group**	**Reads (R1+R2)**	**Total alignments**	**Contacts**
SM427	RP1	478,954,372	520,726,936	84,571,635
SM427	RP2	474,518,690	463,729,357	66,180,319
SM427*	RP3	1,987,996	21,351,232	4,250,818
CN PIP5Pase Tet +	RP1	474,518,690	450,581,626	70,610,174
CN PIP5Pase Tet +	RP2	584,473,958	568,216,739	116,696,341
CN PIP5Pase Tet +	RP3	432,674,790	472,262,320	111,463,674
CN PIP5Pase Tet -	RP1	510,936,252	498,796,338	85,736,879
CN PIP5Pase Tet -	RP2	475,100,422	514,872,190	101,158,068
CN PIP5Pase Tet -	RP3	337,485,098	366,270,012	68,026,438

Supplementary Table 2. Hi-C statistics of summed biological replicates. The number of cis and trans contacts is also shown. Tet, tetracycline. + = with; - = without.

Samples	Total contacts	Cis-contacts	Trans-contacts
SM427	155,002,772	117,721,249	37,281,523
CN PIP5Pase Tet +	298,770,189	264,102,431	34,667,758
CN PIP5Pase Tet -	254,921,385	210,337,093	44,584,292
