# Supplementary material for: Chromosome compartment assembly is essential for subtelomeric gene silencing in trypanosomes
Source: Nat Commun. 2025 Nov 26;16:11669. doi: 10.1038/s41467-025-66824-3 (PMC12749359; doi:10.1038/s41467-025-66824-3)
Supplement: Supplementary file 2 — Description of Additional Supplementary Files [file 41467_2025_66824_MOESM2_ESM.pdf]

Description of Additional Supplementary Files

Supplementary Data 1. Proteins identified by XLMS of PIP5Pase-V5 immunoprecipitation.
Total proteins identified by XLMS of PIP5Pase-V5 immunoprecipitations with anti-V5 monoclonal antibodies. The data show combined results from 11 biological replicates with an FDR of 1%.

Supplementary Data 2. Enrichment analysis of PIP5Pase-V5 immunoprecipitations.
Enrichment analysis of PIP5Pase-V5 immunoprecipitations (11 biological replicates) with anti-V5 monoclonal antibodies compared to immunoprecipitations in cells that do not express V5-tagged PIP5Pase (4 biological replicates). The data shows a complete list of proteins and a subset of enriched proteins (log2 fold-change ≥ 2, *p*-value ≤ 0.05) selected based on their co-immunoprecipitations with PIP5Pase from previous mass spectrometry data [13, 33]. All peptides identified for a protein were used for the analysis, comparing peptide spectral matches between groups.

Supplementary Data 3. Nuclear proteins identified by XLMS of PIP5Pase-V5 immunoprecipitations.
Nuclear proteins identified by XLMS of PIP5Pase-V5 immunoprecipitations with anti-V5 monoclonal antibodies. The data show combined results from 11 biological replicates with an FDR of 1%.

Supplementary Data 4. PIP5Pase-V5 direct and counterpart interactions identified by XLMS.
The data show the complete list of cross-linked proteins, as well as multiple cross-linked proteins (i.e., proteins cross-linking with more than 6 proteins or between 3 and 6 proteins). The data show combined results from 11 biological replicates with an FDR of 1%.

Supplementary Data 5. *In vitro* cross-linked proteins identified after PIP5Pase-V5 immunoprecipitation.
Proteins identified after PIP5Pase-V5 immunoprecipitation with anti-V5 monoclonal antibodies followed by *in vitro* cross-linking. The immunoprecipitated proteins were cross-linked *in vitro* with 0.25 mM of DSS for 10 minutes, quenched with 50 mM glycine before digestion and mass spectrometry analysis. The data show results with 1% FDR.

Supplementary Data 6. Analysis of TADs and loops from Hi-C and Pore-C data.
The data shows TADs identified searching Hi-C or Pore-C matrices at 1Kb, 10 Kb, and 50 Kb resolutions. It also shows all merged TADs from Hi-C data, all merged TADs from Pore-C data, and all merged TADs from Hi-C and Pore-C data. TAD relatedness is also shown, i.e., TADs which are found within overlapping regions. The data also includes the list of loops identified in the Hi-C data using a 10 Kb resolution matrix.
