# Supplementary material for: Chromosome compartment assembly is essential for subtelomeric gene silencing in trypanosomes
Source: Nat Commun. 2025 Nov 26;16:11669. doi: 10.1038/s41467-025-66824-3 (PMC12749359; doi:10.1038/s41467-025-66824-3)
Supplement: Supplementary file 9 — Reporting Summary [file 41467_2025_66824_MOESM9_ESM.pdf]

nature portfolio

Corresponding author(s): Igor Cestari

Last updated by author(s): Nov 12, 2025

Reporting Summary

Nature Portfolio wishes to improve the reproducibility of the work that we publish. This form provides structure for consistency and transparency in reporting. For further information on Nature Portfolio policies, see our Editorial Policies and the Editorial Policy Checklist.

Statistics

For all statistical analyses, confirm that the following items are present in the figure legend, table legend, main text, or Methods section.

n/a	Confirmed	
☐	☒	The exact sample size (*n*) for each experimental group/condition, given as a discrete number and unit of measurement
☐	☒	A statement on whether measurements were taken from distinct samples or whether the same sample was measured repeatedly
☐	☒	The statistical test(s) used AND whether they are one- or two-sided<br>*Only common tests should be described solely by name; describe more complex techniques in the Methods section.*
☒	☐	A description of all covariates tested
☐	☒	A description of any assumptions or corrections, such as tests of normality and adjustment for multiple comparisons
☐	☒	A full description of the statistical parameters including central tendency (e.g. means) or other basic estimates (e.g. regression coefficient) AND variation (e.g. standard deviation) or associated estimates of uncertainty (e.g. confidence intervals)
☐	☒	For null hypothesis testing, the test statistic (e.g. *F*, *t*, *r*) with confidence intervals, effect sizes, degrees of freedom and *P* value noted<br>*Give P values as exact values whenever suitable.*
☒	☐	For Bayesian analysis, information on the choice of priors and Markov chain Monte Carlo settings
☒	☐	For hierarchical and complex designs, identification of the appropriate level for tests and full reporting of outcomes
☒	☐	Estimates of effect sizes (e.g. Cohen's *d*, Pearson's *r*), indicating how they were calculated

Our web collection on statistics for biologists contains articles on many of the points above.

Software and code

Policy information about availability of computer code

Data collection	na
Data analysis	Raw fastq files were mapped to genome using bwa-mem version 0.7.18 or minimap2 version 2.28. Hi-C or Pore-C contacts were obtained using PairTools version 1.1.3. Alignments were analyzed with SAMtools version 1.21. Pair files were converted to cool files using cooler version 0.10.2. The matrices of three biological replicates were combined and corrected using HiCExplorer version 3.7.6. TADs, loops, and Hi-C normalization and group comparisons were performed with HiCExplorer version 3.7.6. FAN-C version 0.9.28 was used for compartment analysis. RNA-seq comparison between groups were performed with EdgeR version 4.8.0. ChIP-seq was analyzed with deepTools version 3.5.6 and MACS 3.0.3, and visualized with Integrated Genomics Viewer tool version 2.17.4. To identify motifs in boundary sequences, we analyzed sequences using Multiple Em for Motif Elicitation (MEME) tool version 5.5.8. Cross-links were analyzed using Kojak version 2.0.3 and validated with Percolator release rel-3-07-01. Surface plasmon resonance was analysed with Traceviewer software version 4.5.8846.19906 (Nicoya). Statistical analysis (t-test) and data graphics were obtained with OriginPro version 2024b. Protein interactions were visualized using Cytoscape 3.9.1, ProxL 2.4.2, and XlinkCyNET 1.5.3. Hi-C, RNA-seq, and ChIP-seq graphs were obtained using HiCExplorer version 3.7.6 or pyGenomeTracks version 3.9. Custom codes are available at https://github.com/cestari-lab/lab_scripts and at Zenodo (10.5281/zenodo.17137491).

For manuscripts utilizing custom algorithms or software that are central to the research but not yet described in published literature, software must be made available to editors and reviewers. We strongly encourage code deposition in a community repository (e.g. GitHub). See the Nature Portfolio guidelines for submitting code & software for further information.

Data

Policy information about availability of data

All manuscripts must include a data availability statement. This statement should provide the following information, where applicable:

- Accession codes, unique identifiers, or web links for publicly available datasets
- A description of any restrictions on data availability
- For clinical datasets or third party data, please ensure that the statement adheres to our policy

RNA-seq and ChIP-seq sequencing data are available in the Sequence Read Archive (SRA) with the BioProject identification PRJNA934938 [https://www.ncbi.nlm.nih.gov/bioproject/934938]. Hi-C sequencing data is available in the SRA with BioProject identification PRJNA1198910 [https://www.ncbi.nlm.nih.gov/bioproject/?term=PRJNA1198910]. The mass spectrometry proteomics data have been deposited in the ProteomeXchange Consortium via the PRIDE partner repository with the dataset identifiers PXD059635 [https://www.ebi.ac.uk/pride/archive/projects/PXD059635]. Codes used for data analysis are available at https://github.com/cestari-lab/lab_scripts and zenodo (https://zenodo.org/records/17137491). Source data is also available with this paper.

Research involving human participants, their data, or biological material

Policy information about studies with human participants or human data. See also policy information about sex, gender (identity/presentation), and sexual orientation and race, ethnicity and racism.

Reporting on sex and gender	na
Reporting on race, ethnicity, or other socially relevant groupings	na
Population characteristics	na
Recruitment	na
Ethics oversight	na

Note that full information on the approval of the study protocol must also be provided in the manuscript.

Field-specific reporting

Please select the one below that is the best fit for your research. If you are not sure, read the appropriate sections before making your selection.

[x] Life sciences [ ] Behavioural & social sciences [ ] Ecological, evolutionary & environmental sciences

For a reference copy of the document with all sections, see nature.com/documents/nr-reporting-summary-flat.pdf

Life sciences study design

All studies must disclose on these points even when the disclosure is negative.

Sample size	Experiments (Hi-C, ChIP-seq, RNA-seq, binding kinetics) were performed in three biological replicates which is the standard in the field to determine variability in dataset. Cross-link and mass spectrometry were performed in 11 biological replicates to increase reproducibility because mass spectrometry data can be variable. Immunofluorescence were performed with three biological replicates. Western blots and SDS/PAGEs for XLMS data were performed for each biological replicate (n = 11). Surface plasmon resonance were performed with three biological replicates.
Data exclusions	none
Replication	Experiment conditions were optimized to for reproducibility. Three biological replicates (or more, as stated above) were performed per experiments.
Randomization	Not applicable
Blinding	No applicable for the type of experiments performed.

Reporting for specific materials, systems and methods

We require information from authors about some types of materials, experimental systems and methods used in many studies. Here, indicate whether each material, system or method listed is relevant to your study. If you are not sure if a list item applies to your research, read the appropriate section before selecting a response.

Materials & experimental systems

n/a	Involved in the study
☐	☒ Antibodies
☐	☒ Eukaryotic cell lines
☐	☐ Palaeontology and archaeology
☐	☐ Animals and other organisms
☐	☐ Clinical data
☐	☐ Dual use research of concern
☐	☐ Plants

Methods

n/a	Involved in the study
☐	☒ ChIP-seq
☐	☐ Flow cytometry
☐	☐ MRI-based neuroimaging

Antibodies

Antibodies used

Monoclonal antibodies (mAbs) α-V5 (ABclonal Inc., catalog # AE017; 1:5000 dilution), mAbs α-HA (ABclonal, catalog # AE065, 1:5000 dilution), mAbs α-HA 3F10 (Invitrogen, catalog # 26183; 1:500 dilution), goat α-mouse IgG (H+L)–Alexa Fluor 488 (Thermo Fisher Scientific, catalog # A11001; 1:1000 dilution), goat anti-mouse IgG (H+L)-HRP (Life Technologies, catalog # 31430; 1:2000 dilution).

Validation

Antibodies were validated in cell lines expressing V5-tagged or HA-tagged proteins in T. brucei cells expressing the tagged proteins induced by tetracycline. Validation was performed by Western blot and immunofluorescence (Touray et al. 2023, eLife; Cestari et al. 2019, Mol Cell Biol).

Eukaryotic cell lines

Policy information about cell lines and Sex and Gender in Research

Cell line source(s)

Trypanosoma brucei Lister 427 strain single-marker (SM427) was obtained from Dr. Ken Stuart laboratory (Seattle Children's).

Authentication

We sequenced the transcriptome and genome (Touray et al. 2023, eLife, and this work) of the cell and compared to the reference genome of the cell lines. The SM427 grows under selection of antibiotic G418; whereas conditional null PIP5Pase in G418, phelomycin, hygromycin, blasticidin, and tetracyclin. We confirmed the genetic cell lines by real-time PCR and DNA-seq.

Mycoplasma contamination

Tested by PCR kit once a year.

Commonly misidentified lines
(See ICLAC register)

Not applicable.

Palaeontology and Archaeology

Specimen provenance

na

Specimen deposition

na

Dating methods

na

☐ Tick this box to confirm that the raw and calibrated dates are available in the paper or in Supplementary Information.

Ethics oversight

na

Note that full information on the approval of the study protocol must also be provided in the manuscript.

Animals and other research organisms

Policy information about studies involving animals; ARRIVE guidelines recommended for reporting animal research, and Sex and Gender in Research

Laboratory animals

na

Wild animals

na

Reporting on sex

na

Field-collected samples

na

Ethics oversight

na

Note that full information on the approval of the study protocol must also be provided in the manuscript.

Clinical data

Policy information about clinical studies

All manuscripts should comply with the ICMJE guidelines for publication of clinical research and a completed CONSORT checklist must be included with all submissions.

Clinical trial registration	na
Study protocol	na
Data collection	na
Outcomes	na

Dual use research of concern

Policy information about dual use research of concern

Hazards

Could the accidental, deliberate or reckless misuse of agents or technologies generated in the work, or the application of information presented in the manuscript, pose a threat to:

No	Yes	
☒	☐	Public health
☒	☐	National security
☒	☐	Crops and/or livestock
☒	☐	Ecosystems
☒	☐	Any other significant area

Experiments of concern

Does the work involve any of these experiments of concern:

No	Yes	
☒	☐	Demonstrate how to render a vaccine ineffective
☒	☐	Confer resistance to therapeutically useful antibiotics or antiviral agents
☒	☐	Enhance the virulence of a pathogen or render a nonpathogen virulent
☒	☐	Increase transmissibility of a pathogen
☒	☐	Alter the host range of a pathogen
☒	☐	Enable evasion of diagnostic/detection modalities
☒	☐	Enable the weaponization of a biological agent or toxin
☒	☐	Any other potentially harmful combination of experiments and agents

Plants

Seed stocks	na
Novel plant genotypes	na
Authentication	na

ChIP-seq

Data deposition

- [x] Confirm that both raw and final processed data have been deposited in a public database such as GEO.
- [x] Confirm that you have deposited or provided access to graph files (e.g. BED files) for the called peaks.

Data access links<br>*May remain private before publication.*	ChIP-seq data from SRA: https://dataview.ncbi.nlm.nih.gov/object/PRJNA934938
Files in database submission	na
Genome browser session<br>(e.g. UCSC)	na

Methodology

Replicates	Three biological replicates
Sequencing depth	Information is available in Touray et al., 2023 eLife (doi: 10.7554/eLife.89331.4).
Antibodies	mAbs α-HA 3F10 (Invitrogen)
Peak calling parameters	macs3 callpeak -t ChIP_Bio1_sorted.bed ChIP_Bio2_sorted.bed ChIP_Bio3_sorted.bed \ -c Input_Bio1_sorted.bed Input_Bio2_sorted.bed Input_Bio3_sorted.bed \ -f BED -g 35000000 -n chip-macs3 --nomodel --extsize 500 --fix-bimodal \ --broad --broad-cutoff 0.05 --outdir path/to/files/macs/
Data quality	FDR = 0.05, fold-enrichment 2
Software	macs3

Flow Cytometry

Plots

Confirm that:

- [ ] The axis labels state the marker and fluorochrome used (e.g. CD4-FITC).
- [ ] The axis scales are clearly visible. Include numbers along axes only for bottom left plot of group (a 'group' is an analysis of identical markers).
- [ ] All plots are contour plots with outliers or pseudocolor plots.
- [ ] A numerical value for number of cells or percentage (with statistics) is provided.

Methodology

Sample preparation	na
Instrument	na
Software	na
Cell population abundance	na
Gating strategy	na

- [ ] Tick this box to confirm that a figure exemplifying the gating strategy is provided in the Supplementary Information.

Magnetic resonance imaging

Experimental design

Design type	na
Design specifications	na
Behavioral performance measures	na

Acquisition

Imaging type(s)	na
Field strength	na
Sequence & imaging parameters	na
Area of acquisition	na

Diffusion MRI ☐ Used ☐ Not used

Preprocessing

Preprocessing software	na
Normalization	na
Normalization template	na
Noise and artifact removal	na
Volume censoring	na

Statistical modeling & inference

Model type and settings	na
Effect(s) tested	na

Specify type of analysis: ☐ Whole brain ☐ ROI-based ☐ Both

Statistic type for inference<br>(See Eklund et al. 2016)	na
Correction	na

Models & analysis

n/a	Involved in the study
☐	☐ Functional and/or effective connectivity
☐	☐ Graph analysis
☐	☐ Multivariate modeling or predictive analysis

Functional and/or effective connectivity	na
Graph analysis	na
Multivariate modeling and predictive analysis	na
