# Supplementary figures and images for: Chromosome compartment assembly is essential for subtelomeric gene silencing in trypanosomes
Source: Nat Commun. 2025 Nov 26;16:11669. doi: 10.1038/s41467-025-66824-3 (PMC12749359; doi:10.1038/s41467-025-66824-3)

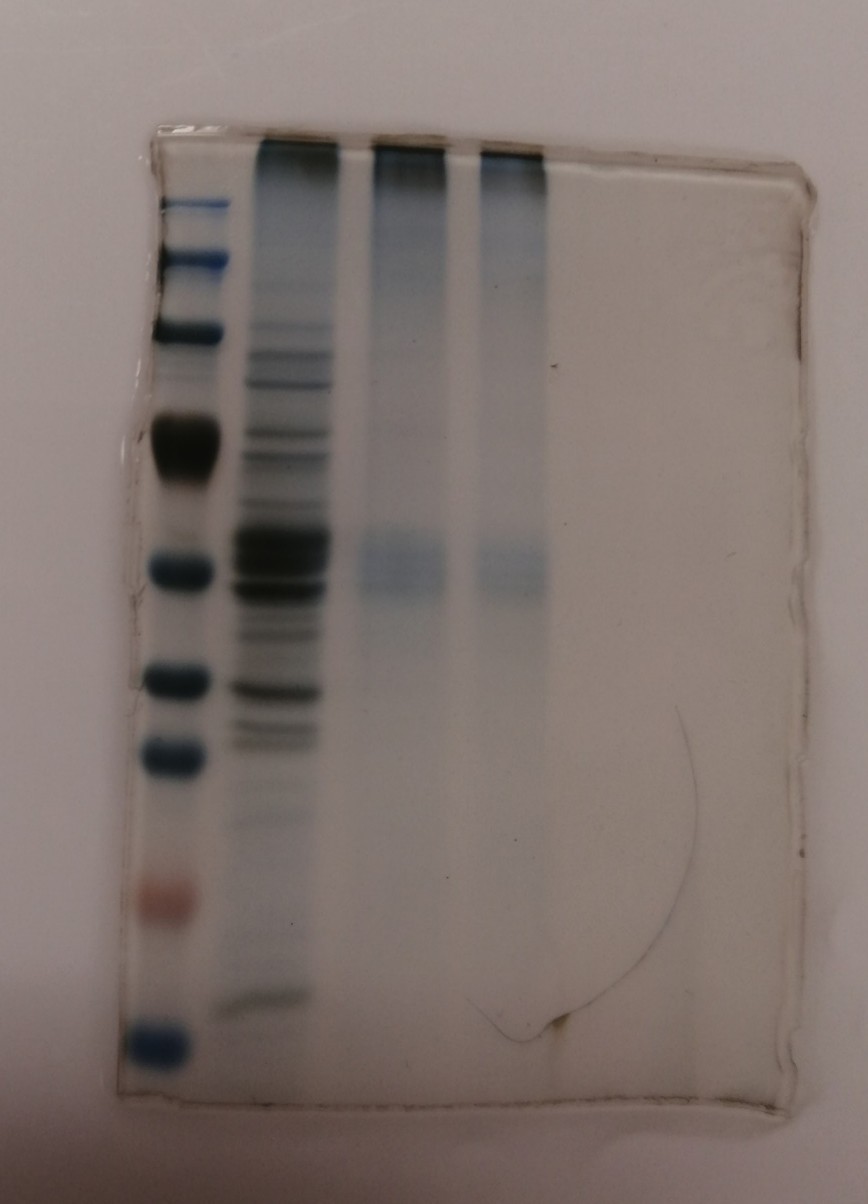

Supplement: Supplementary file 11 — Source data [file 41467_2025_66824_MOESM11_ESM.zip › Source_data/Figure 1B.jpg]

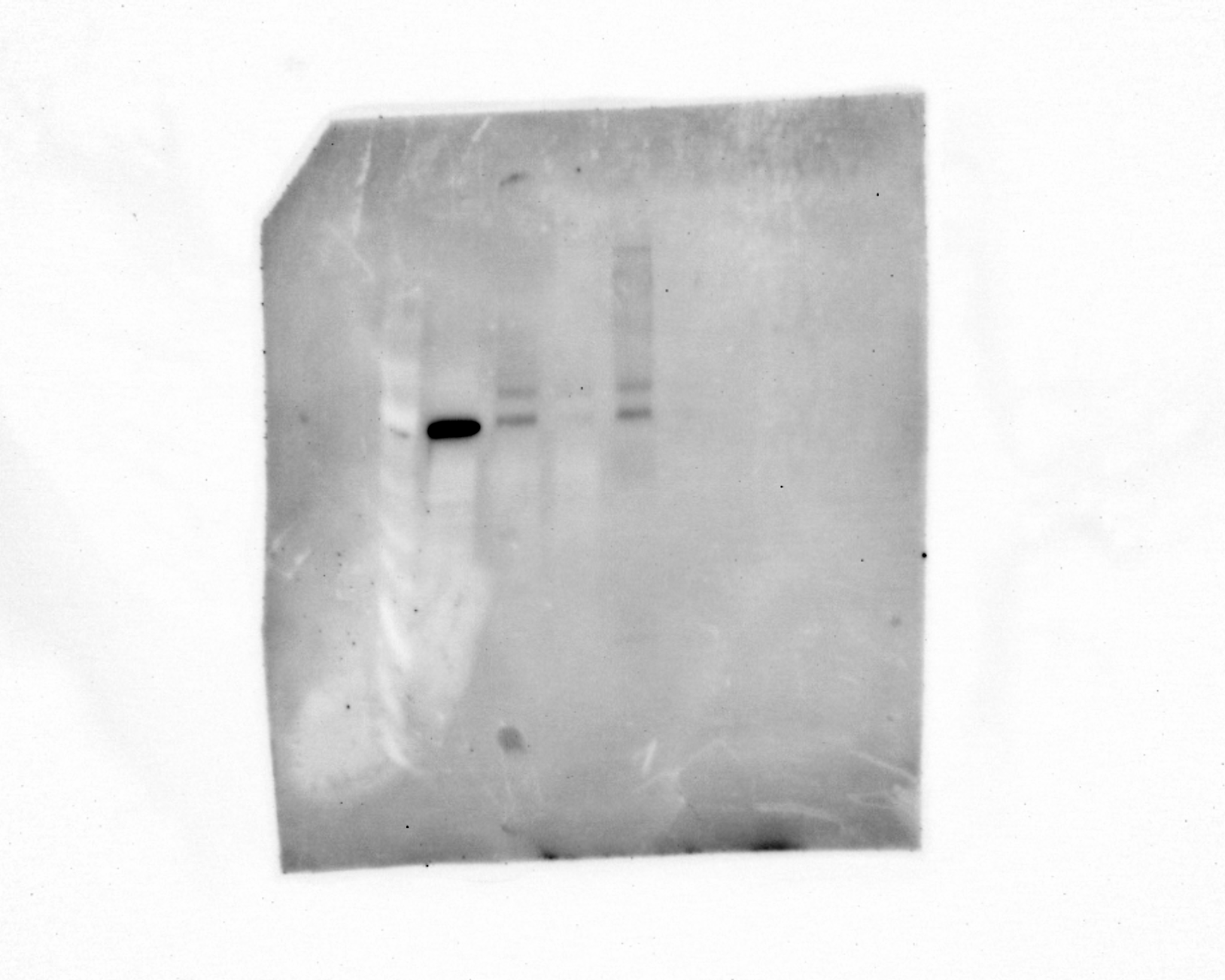

Supplement: Supplementary file 11 — Source data [file 41467_2025_66824_MOESM11_ESM.zip › Source_data/Figure 1C.tif]

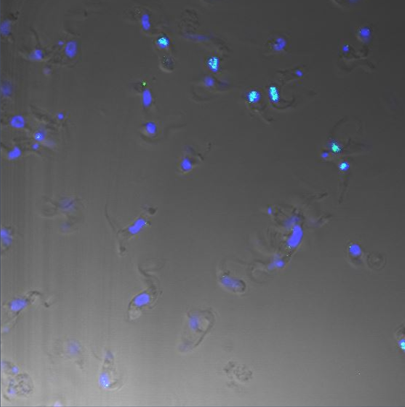

Supplement: Supplementary file 11 — Source data [file 41467_2025_66824_MOESM11_ESM.zip › Source_data/Mut - DIC-M.png]

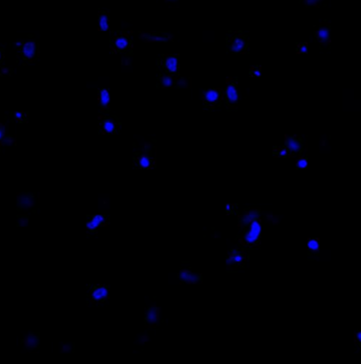

Supplement: Supplementary file 11 — Source data [file 41467_2025_66824_MOESM11_ESM.zip › Source_data/Mut-DAPI.png]

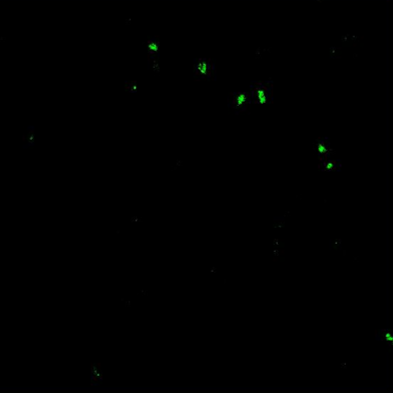

Supplement: Supplementary file 11 — Source data [file 41467_2025_66824_MOESM11_ESM.zip › Source_data/Mut-RAP1-HA.png]

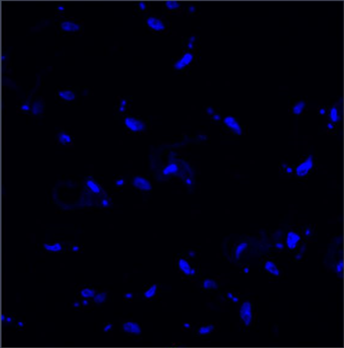

Supplement: Supplementary file 11 — Source data [file 41467_2025_66824_MOESM11_ESM.zip › Source_data/WT - DAPI.png]

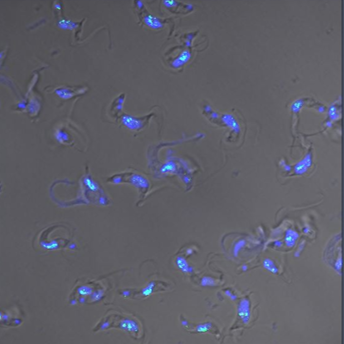

Supplement: Supplementary file 11 — Source data [file 41467_2025_66824_MOESM11_ESM.zip › Source_data/WT - DIC-M.png]

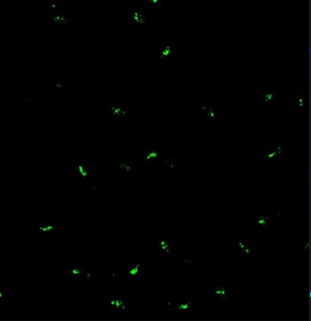

Supplement: Supplementary file 11 — Source data [file 41467_2025_66824_MOESM11_ESM.zip › Source_data/WT - MERGE.png]

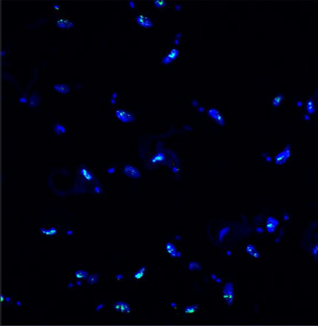

Supplement: Supplementary file 11 — Source data [file 41467_2025_66824_MOESM11_ESM.zip › Source_data/WT - RAP1-HA.png]
